# Insole-Based Systems for Health Monitoring: Current Solutions and Research Challenges

**DOI:** 10.3390/s22020438

**Published:** 2022-01-07

**Authors:** Sophini Subramaniam, Sumit Majumder, Abu Ilius Faisal, M. Jamal Deen

**Affiliations:** 1 School of Biomedical Engineering, McMaster University, Hamilton, ON L8S 4L8, Canada; subras1@mcmaster.ca; 2 Electrical and Computer Engineering, McMaster University, Hamilton, ON L8S 4L8, Canada; majums3@mcmaster.ca (S.M.); faisaa4@mcmaster.ca (A.I.F.); 3 Department of Biomedical Engineering, Chittagong University of Engineering and Technology, Chattogram 4349, Bangladesh

**Keywords:** health monitoring, plantar pressure distribution, gait analysis, wearable sensors, medical device, smart insole, pressure sensor, IMU

## Abstract

Wearable health monitoring devices allow for measuring physiological parameters without restricting individuals’ daily activities, providing information that is reflective of an individual’s health and well-being. However, these systems need to be accurate, power-efficient, unobtrusive and simple to use to enable a reliable, convenient, automatic and ubiquitous means of long-term health monitoring. One such system can be embedded in an insole to obtain physiological data from the plantar aspect of the foot that can be analyzed to gain insight into an individual’s health. This manuscript provides a comprehensive review of insole-based sensor systems that measure a variety of parameters useful for overall health monitoring, with a focus on insole-based PPD measurement systems developed in recent years. Existing solutions are reviewed, and several open issues are presented and discussed. The concept of a fully integrated insole-based health monitoring system and considerations for future work are described. By developing a system that is capable of measuring parameters such as PPD, gait characteristics, foot temperature and heart rate, a holistic understanding of an individual’s health and well-being can be obtained without interrupting day-to-day activities. The proposed device can have a multitude of applications, such as for pathology detection, tracking medical conditions and analyzing gait characteristics.

## 1. Introduction

There is a growing demand for low-cost, unobtrusive and reliable wearable healthcare solutions to meet the increasing demand for healthcare services that are progressively more expensive due to soaring costs of diagnostic tools, in-clinic care, and prescription drugs [1]. Such wearable solutions may enable long-term telemonitoring of individuals with limited access to healthcare services or living under fixed budget conditions. These solutions can potentially enable early diagnoses and interventions of diseases and reduce the frequency of visits to or long-term stays at expensive healthcare facilities [2,3]. Economical and smart devices for healthcare were largely made possible due to recent progress with miniaturized sensors and actuators as well as advances in wireless communication, computing and information technologies [3,4,5]. An insole-based foot health monitoring system is such a solution that can allow for the overall status of an individual’s health to be monitored in a cost-effective and simple manner during day-to-day life.

The foot is used in locomotion activities, bears an individual’s weight and provides balance and support to an individual, which requires proper functioning of the neuromuscular system. In order to maintain healthy and normal gait, it is essential for organ systems such as the cardiovascular, respiratory, nervous and musculoskeletal systems to function properly [3,6]. Thus, it is recognizable that gait and foot health is related to the overall condition of an individual’s health [3,7]. For example, people with diabetic peripheral neuropathy (DPN) tend to have a higher foot plantar pressure [8], thus resulting in a higher risk for plantar ulceration [9]. On the other hand, individuals with early-stage Parkinson’s disease were shown to have smaller, shuffling steps [5,10]. Additionally, these individuals periodically have trouble with starting, stopping, turning and picking up their feet while walking [3,11,12]. Thus, a device for monitoring and quantitatively assessing foot health can be of immense use for identifying anomalies in plantar pressure, activity and gait characteristics, particularly at early stages of disease or during rehabilitation of lower-limb illnesses or injuries.

Accelerated progress in micro-electro-mechanical systems (MEMS) technologies has allowed for the development of sensors and actuators that have high sensitivities, are miniaturized and inexpensive. Wearable solutions which incorporate pressure sensors can greatly enhance health monitoring applications while also ameliorating interactions between humans and technology [13]. In addition, inertial measurement devices which are MEMS–based, such as accelerometers and gyroscopes, are being incorporated into numerous applications such as trackers for fitness, smartphones and vehicles [3,14,15]. In fact, in the era of the internet-of-things (IoT) and internet-of-everything (IoE) it is anticipated that such small-sized actuators and sensors will have a crucial role in smart systems and smart devices [6,16]. Therefore, wearable pressure sensors and inertial measurement units (IMUs), coupled with the high-speed communication and computing technologies of current smart devices can be used for real-time monitoring of plantar pressure, activity and gait pattern, and for performing a quantitative assessment of foot health in the day-to-day activities of the individuals. Furthermore, insole-based and related systems can potentially be used to realize a comprehensive health monitoring tool by integrating additional sensors for monitoring heart rate [17,18], temperature [19], and muscle activity [20]. The non-obtrusive nature of such a system also results in gait being minimally impacted, allowing for the measurements obtained to be more reflective of our natural gait.

To transmit data to a computing platform in close proximity, such smart insoles can employ a range of short-range technologies. For instance, near field communication (NFC), ANT protocol, Bluetooth, Bluetooth low energy (BLE) or ZigBee can be used [2,11,20,21]. Furthermore, a smart insole, when coupled with modern-day long-range wireless communication technologies such as high-speed packet access (HSPA), and long-term evolution (LTE) services, is able to provide uninterrupted and high-speed internet connectivity to any location [1,3,9]. Such a smart insole can potentially create a pathway towards unobtrusive remote health monitoring, which can allow for healthcare professionals such as clinicians, physicians and formal caregivers, to oversee and evaluate a person’s health data, even from distant locations. 

**Figure 1:**
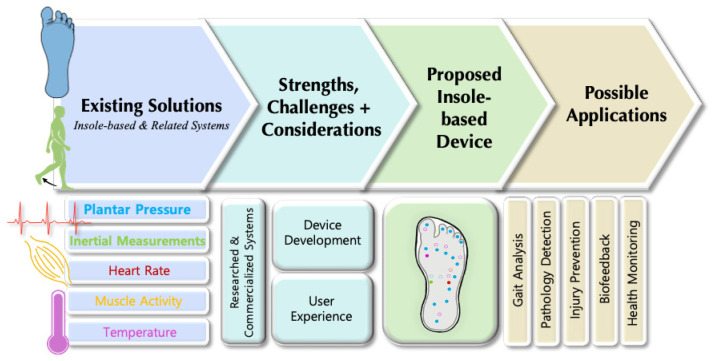
Overview of review article.

In this article, we present a detailed review of the current state of research and development in the insole-based systems for monitoring plantar pressure, activity and gait, in addition to addressing associated challenges and examining how current solutions can be improved (Figure 1). After the Introduction (Section 1), the anatomical background for plantar pressure distribution (PPD) measurements and the motivation behind using insole-based sensor systems for monitoring PPD, activity and gait are described in Section 2. In Section 3, an overview of insole-based health monitoring systems is provided. This section includes research systems from the recent literature including triboelectric sensors, as well as commercial systems. Although, particular attention was on gait, activity and plantar pressure monitoring aspects of insole-based systems, we also discussed several other systems that reported monitoring of other health parameters such as heart rate (HR), muscle activity and body temperature. Then in Section 4, a discussion on different engineering considerations in terms of signal integrity, signal processing, data analysis, pattern recognition and prediction is presented. In Section 5, the challenges associated with the development of such systems and their potential solutions, as well as a discussion on future research perspectives is given. In future perspectives, we included a proposed insole-based sensor system for holistic health monitoring, in addition to outlining other considerations that should be taken to improve the smart insole development and the user experience. Then in Section 6, we summarize the major benefits and potential applications of using insole-based sensor systems for health monitoring. 

## 2. Motivation

Continuous monitoring of physiological parameters in a nonobtrusive and automatic manner during daily living allows for potential pathologies to be detected at the very onset, and thus can be immensely beneficial in improving an individual’s quality of life. Comprehensive health monitoring in our home environment also has important benefits over lab-based testing, as the measurements are reflective of our health in natural living conditions. In addition, measurements can be obtained continuously or repeatedly at different times of the day, thus further facilitating long-term tracking of medical condition, diseases progression and rehabilitation. Such a monitoring system, when embedded in the insole can be immensely beneficial in obtaining information regarding an individual’s PPD (PPD), gait characteristics, heart rate and foot temperature. These parameters can provide insights into an individual’s overall health and indicate the presence or onset of a particular pathology for further investigations. Furthermore, measuring these parameters synchronously in a single system can allow for more meaningful assessment of an individual’s health as associations and assessment relationships between different parameters can be determined.

### 2.1. Plantar Pressure Distribution

**Figure 2:**
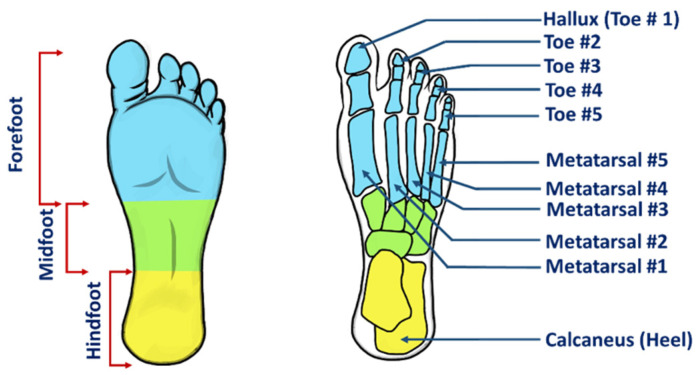
Foot regions commonly investigated in PPD studies.

In 2020, 18% of Canadians were over the age of 65 [22] and this number continues to rise. There has also been nearly a 12% increase in the number of Canadians above the age of 12 who have diabetes, from 2018 to 2019 [23]. From 2018 to 2019, the prevalence of obesity and arthritis have also increased in Canada [24]. As the aging population continues to rise, the prevalence of pathologies present in individuals worldwide is also increasing. Foot and associated pathologies and consequently mobility disability, have an increasing prevalence [25,26]. Several medical conditions are also related to changes in foot health, particularly relating to an individual’s PPD [27,28]. The proportion of individuals who may develop foot problems is growing, and this can have tremendous social and economic impacts. In 2010, it was determined that 15% of Canadians with diabetes were likely to develop a diabetic foot ulcer at some point in their lifetime [29]. With the incidence of diabetes on the rise, the number of individuals with diabetic foot ulcers and other foot complications is also expected to rise. As individuals engage in various activities and more hectic lifestyles, the distribution of pressure across the foot, and foot health in general, may not be appropriately monitored. Small changes in an individual’s posture and deviations in an individual’s foot structure or PPD can both be indicative of pathologies as well contribute to pathologies, such as lower limb joint and muscle problems. Groups of individuals which have characteristic PPD patterns include older adults, individuals with pes cavus and pes planus, diabetes, hip and knee osteoarthritis, obesity, autism and those who engage in athletic activities. A visual depiction of the regions of the foot that are typically investigated in PPD studies is shown in Figure 2. 

Monitoring of foot PPD can be beneficial in improving health and treating physiological conditions, including foot deformities such as pes cavus and pes planus. This is often done through the use of custom orthotics or addressing the cause that leads to abnormal gait, such as having hip pain which may impact walking or foot health. Information regarding PPD can be particularly useful for designing custom orthotics in order to better support specific regions of the foot, which can reduce the risk of falling or developing adverse foot or lower extremity pathologies. Abnormalities in the distribution of plantar pressure or gait has been associated with adverse long-term health effects, such as having an increased slip and fall risk [30,31], diabetes [32], or neurodegenerative diseases [33,34], as well as anxiety and depression [35], and thus can be used as a diagnostic indicator to signal for such physiological or psychological abnormalities [35]. For instance, individuals with hip osteoarthritis tend to exert less pressure on the heel than those without hip osteoarthritis [36]. Observations as such prove to be beneficial for the indication of physiological issues which can be further investigated and addressed to prevent further damage to other body parts, such as muscles and joints. Differences in PPD have also been observed in the gait characteristics of autistic children compared to their non-autistic counterparts [37,38]. Therefore, foot pathologies and associated medical conditions can potentially be detected at their early onset through regular monitoring of an individual’s PPD, thereby reducing the long-term impacts of abnormal gait and PPD. Further, the benefits of analyzing an individual’s PPD can span beyond improving foot health. In fact, having an improved gait was shown to be associated with improved overall health [31]. Additionally, correcting PPD to be closer to healthier values can reduce muscle pain [39]. Identifying abnormal gait patterns and attempting to correct or adjust for deviations from normal can be used to improve the quality of life and the health of individuals, and of older adults in particular [40].

Common gait analysis methods include using force platforms [41] and motion capture technology [42]. Although such technologies provide detailed information regarding an individual’s PPD or gait characteristics, respectively, they also have some limitations. Such limitations include the extensive set-up of equipment, using the technology in laboratory-based environments which are not in the user’s natural setting, having shorter durations of measurements taken, and potential inconveniences to users who must arrange a separate time to have measurements taken. On the other hand, insole-based sensor systems allow for non-obtrusively monitoring an individual’s PPD during their daily activities, allowing one to maintain their natural gait during measurements. These systems are gaining prevalence due to their low development cost and high measurement accuracies of various step phases [43,44]. Such systems can be used in diverse environments and conditions, including during the user’s daily activities and in their natural setting, and can allow for monitoring an individual’s PPD for extended time periods, such as when used in all-day wear. It can further facilitate measuring ground reaction force and ankle moment that are highly correlated with gait balance and stability [41].

### 2.2. Gait and Activity Monitoring

For humans, walking is the main method of locomotion and a critical aspect of physical activity. Walking is a periodic-type movement that includes complicated cyclic motions of the lower limbs and requires proper functioning and coordination of the nervous, musculoskeletal and cardiorespiratory systems [45]. The word “gait” describes an individual’s way of walking, and the gait pattern is highly associated with the overall health condition [46]. Furthermore, gait patterns vary among individuals of different demographic and physical characteristics. For example, elderly individuals with abnormal gait were shown to have an increased fall risk [47], and gait speeds below 60 cm/s is associated with poor mobility and health [31]. Gait analysis allows for assessing stability and balance during walking and is employed extensively in several health and mobility-related applications such as orthopedics, rehabilitation, health diagnostics, and sports. In addition, gait analysis can potentially be used in the early detection of neurodegenerative diseases such as Parkinson’s and Alzheimer’s [48].

Currently, there are several gait analysis techniques and devices available that can monitor and record information related to gait. Based on the analysis techniques and sensor technologies, these gait analysis methods can be divided into three categories: (1) Image processing (IP)-based [49,50,51,52,53,54]; (2) floor sensors (FS)-based [55,56,57,58]; and (3) wearable sensors (WS)-based [12,13,14,15,16,17,18,19,20,21] which are worn and carried by the user [59]. The IP-based system captures image data of the subject’s gait through one or multiple optical sensors and extracts gait information through digital image processing [49,50,51,52,53,54]. Digital cameras are the most commonly used devices in such systems. However, other types of optical sensors such as time-of-flight (TOF) cameras [60,61], infrared (IR) sensors [62], and laser range scanners (LRS) [63] are also used. On the other hand, in FS-based systems, force or pressure sensors are placed on the floor [55,56,57,58]. These types of systems are known as force platforms or instrumented walkways where gait is measured by pressure or force sensors and moment transducers when a person walks on them. Although both systems are considered reliable and widely used for gait analysis, they require complex and expensive infrastructures, pre-equipped environment and setup that restricts users’ usual movements and makes the system incompatible for real-time or regular gait and activity analysis [59]. In addition, FS-systems are unable to provide any information during the swing phase of the gait cycle, thus they only have limited application.

In order to overcome these limitations, researchers have been focusing on wearable sensors (WS)-based gait and activity monitoring systems [12,13,14,15,16,17,18,19,20,21]. Modern miniature and lightweight wearable sensors with wireless connectivity have made it possible to develop user-friendly gait analysis systems and to obtain real-time gait data without hindering the user’s regular day-to-day activities. These sensors are mounted on different body parts, such as the feet, knee, thigh, waist, or shoulder, to measure the movements of the human body. Different types of wearable sensors are used to capture gait signals such as flexible goniometers, ultrasonic sensors, electromyography (EMG) sensors, inertial measurement units (IMUs), textile-based force sensors, acoustic sensors, and optical sensors. Among the wearable sensor technologies, IMUs are the most promising sensor systems for analyzing movements during gait because of their small size and capability to measure three-dimensional motion and orientation with high precision and accuracy [64,65,66,67,68]. Therefore, a combination of pressure sensors and IMUs embedded in the insole may potentially enable comprehensive gait analyses by detecting simultaneous motion and plantar pressure during walking.

## 3. Insole-Based Monitoring Systems

### 3.1. Research Systems

**Figure 3:**
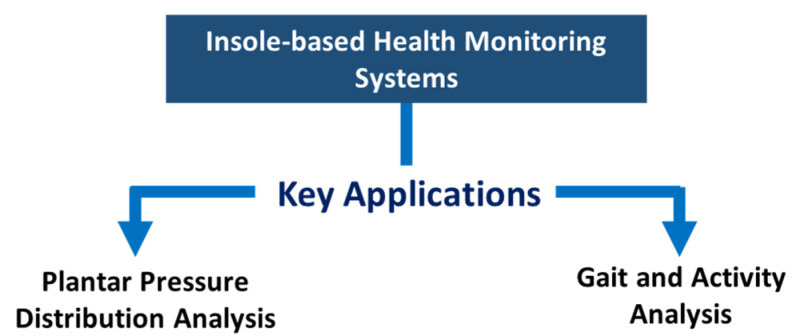
Insole-based systems typically have one of two focuses: obtaining detailed PPD or gait event/characteristic analysis.

Several insole-based health monitoring systems were proposed in the literature. However, based on the application and sensing mechanism, these monitoring systems can be broadly categorized into two types (Figure 3)—foot pressure monitoring systems and gait monitoring systems. The systems for foot pressure measurements use pressure sensors to measure gait event/characteristic or detailed information regarding PPD. On the other hand, the systems for gait monitoring mainly rely on IMUs to measure gait dynamics and to monitor gait pattern and activities. In this section, we present a brief discussion on some published insole-based lower-limb health monitoring systems.

#### 3.1.1. Foot Pressure Monitoring

A wireless insole device in [69] was developed to measure plantar pressure during daily activity, particularly when standing and walking. There, they integrated piezoelectric sensors at 24 points into an insole, with three sensors placed at each of the eight measuring points. Each of the three sensors at a measuring point accounted for the x, y or z axes, in order to take 3-dimensional measurements. The lightweight (10 g) and small size (10 × 20 × 8 mm^3^) of the pressure measurement unit can potentially make it convenient to use with any shoe. In addition, the device is reportedly capable of recording data for over 24 h, making it ideal for full-day use. However, more measuring points along with additional sensors could potentially allow for obtaining the detailed measurement of PPD of the entire foot. It was recommended in [70] to use one sensor at each of 15 anatomically relevant regions of the foot (placed beneath the toes, metatarsals, midfoot and hindfoot that can potentially provide the PPD with sufficient resolution. Three measuring points were at the heel and five were at the forefoot. Additional measuring points placed at the toes, particularly the hallux, in addition to the midfoot, can allow for obtaining more holistic measurements of the foot. There, they used piezoelectric sensors to achieve high sensitivity, high signal-to-noise ratio (SNR), and increased stability with temperature. However, the system was validated on only three males, all 20 years and of similar body weight. It is indeed important to test for repeatability among a specific group of individuals, yet ensuring the measurement consistency of such a device in other members of the general population, such as females and older adults is also critical. Furthermore, pressure distribution at the foot changes with age and differs between males and females due to morphological differences in foot structure. Thus, it is important to identify the optimal location and number of sensors to ensure the accuracy and consistency of plantar pressure measurements in a wide cross-section of individuals.

A soft-strain sensor was developed in [71] to detect pressure-induced deformation that can potentially be used in insole-based systems to monitor various gait parameters such as ground reaction force and calf muscle activity, and several physiological parameters such as respiratory rate (by detecting chest expansion), and heart rate (by detecting pressure changes with arterial blood flow). The sensor was fabricated by incorporating carbon black and carbon fiber conductive particles on a non-conductive silicone matrix. The flexible nature of the soft-strain sensors can provide wearing comfort for an extended period of time, thus making them ideal for long-term monitoring. The researchers reported an error of 1% and 2.5% during walking and running, respectively, when the sensor was used for gait sensing (recording number of steps taken). However, experiments were conducted only on two individuals, and therefore, further research is required to investigate the accuracy of the sensor and its applications. For instance, pressure values at the foot during certain activities (such as when jumping) are much higher than the deformation of the device caused by pressure exerted when breathing. Nevertheless, the base resistance of the sensor can be tailored for the application by changing the amount of carbon fiber and carbon black. The three-dimensional shape of the insole (if used for in-shoe pressure sensing), can be altered by changing the mold as well. Such a system may be particularly useful when designing insoles for diverse individuals as the system’s sensitivity, resistance and shape can be customized to the user.

A graphene-based flexible pressure sensor was developed in [72] to measure plantar pressure and analyze gait. The graphene layers surround individual polyester fibers that show a piezoresistive effect upon deformation with pressure. This sensor is able to measure up to 800 kPa of pressure, thus making it suitable for an insole-based system to measure PPD during daily-life activities such as walking or higher-impact activities. In addition, the sensor showed a linear response and had a fast response time, making it a promising candidate for flexible pressure sensors to be able to sense pressure accurately and quickly in a comfortable and reliable manner. 

To sense plantar pressure, a rubber insole involving capacitive sensors and polydimethylsiloxane (PDMS) was created in [73]. It that work, it was indicated that piezoresistive sensors used for in-shoe plantar pressure measurements have higher dependence on temperature and lower sensitivity compared to capacitive sensors. As a result, this team used printed capacitive pressure sensors integrated in a rubber insole. Specific benefits of the interdigitated capacitor (IDC) used in this system include its low weight, small size and appropriate sensitivities (67 pF/MPa or 4.30 V/MPa) required for such systems. The elastic rubber that the printed interdigitated capacitors are embedded into is PDMS. The use of PDMS in this insole serves two functions: for the encapsulation of the IDCs and as a dielectric medium. The 3D-printed mold of the insole is filled with PDMS and the interfacing circuits, which are cured for two hours at 60 degrees Celsius. The device allows for the capacitance variation to be obtained from the pressure applied. The encapsulation of the sensors by PDMS does have an effect on the capacitance values obtained. Compared to when exposed to air, the sensors encapsulated in PDMS show increased capacitance, which results from an increase in relative permittivity. However, this study found only a 3% difference in the values obtained, likely attributable to air bubbles being present around the capacitors. It is also indicated that such air gaps are important to the sensing capability of this device, as the sensitivity depends on the relative permittivity change, which is affected by the dielectric medium surrounding the sensors. However, the quantity of air gaps present may not necessarily be consistent among different insoles, and such a difference may not be large enough to meaningfully alter the plantar pressure measurements obtained. Although such a sensor can be very useful in developing insoles tailored to specific foot morphologies, one drawback is that only three areas of the foot were investigated: the forefoot, midfoot and hindfoot. One sensor is placed in each of these three regions, which are important to sense and because they show the most variation when engaging in different activities. However, including more sensors would allow for more specific regions of the foot to be investigated. However, this insole device [73] showed differences in pressure among the three regions of the foot when engaging in various postures such as leaning forward, leaning backward, or standing in a natural position. Therefore, it can also be used for gait analysis, or components from this sensor system can be integrated into a more complex in-shoe plantar pressure monitoring system to obtain accurate values for more specific regions of the foot.

In [74], PPD measurements and gait monitoring were accomplished using piezoelectric sensors combined with an IMU in an insole. A firm base was used for the insole, onto which ceramic piezoelectric discs were placed in five locations. An IMU was also placed in the base of the insole at the midfoot region. Using low-cost sensors, this piezo-insole was able to successfully detect key gait events based on pressure distributions at five points on the foot, in addition to the IMU data. In situations where cost is a major factor in providing care, such systems that provide a more affordable solution can be used for gait analysis. However, for more in-depth analysis of PPD, fifteen sensing points should ideally be used [70]. The integration of an IMU can also provide further information in addition to detailed information regarding pressure when the foot contacts the ground. In particular, the IMU data can be used for analysis during the swing phase of the gait cycle, whereas the pressure data can be used for the stance phase. Incorporating IMUs with insole-based systems for plantar pressure measurements can provide important information regarding an individual’s gait patterns and overall health. 

An insole-based system containing optoelectronic sensors designed for assessing gait, particularly for estimating gait phases and recognizing discrete events, such as toe-off and heel-strike, was developed in [75]. The sensors used in this device [75] are based on optoelectronic technology, with each sensor having a light-emitting diode (LED) along with a silicone cover that may be deformed. When pressure is applied to the surface of the sensor, the silicone deforms and reduces the light that passes from the LED emission area to where the light is received. This causes the output voltage to be altered. The device developed in this study had the aim of being simple and low-cost, in order to obtain the minimum information necessary to determine gait events. The system was designed to reduce unnecessary complexity that would otherwise be found in a device with the aim of obtaining detailed information regarding PPD. Fifteen sensors is the ideal number of sensors to be used in an insole-based device, if the sensors cover the 15 key areas of the foot to be included in measurements [70]. Based on this information, 16 sensors were used in the device presented in [75], and these sensors were placed according to the subjects’ walking patterns on a treadmill. While some sensors were clustered in some areas, others were placed at points of the foot where the plantar pressure is highest. Such placement has its advantages and disadvantages, which also depend on the application. An advantage of clustering the sensors is that there is redundancy, which allows for improved reliability of the data obtained. If one sensor becomes damaged or malfunctions, other sensors in the same area can compensate and provide results. Clustering of sensors is particularly useful in devices that are to be used for extended duration (such as for full-day wear) and that are used repeatedly (such as over several weeks or months), as sensor degradation may occur over this time. Although a large number of sensors were used (in accordance with the number of sensors recommended in [70], the placement of the sensors in this study did not necessarily reflect the 15 key anatomical regions of the foot for plantar pressure measurements. For the particular application of recognizing gait events in this study, the limited areas of the foot examined was sufficient. However, for systems that aim to measure plantar pressure in greater detail, additional sensors should be placed at other anatomically relevant areas of the foot. Ultimately, to account for the 15 anatomically relevant areas of the foot and use sensor clustering, more sensors would have to be implemented in the design of the insole. However, drawbacks of this include greater battery power consumption and increasing the cost of the device. As a result, the particular application to be investigated and the amount of redundancy desired should carefully be considered when designing an insole-based system to measure PPD. 

The insole developed in [76] had seven conductive rubber sensors which are sensitive to pressure. This system also used wireless data transmission for nonobtrusive, portable monitoring of daily activities. A particular advantage of this device is that the wireless data transmission unit is small in size (25 × 15 × 8.5 mm^3^) and lightweight (12 g), and the sensors used in this device were 15 × 10 × 0.8 mm^3^ in size. This is particularly important as the smaller and thinner the device is, the less it will impact an individual’s natural gait. The seven insole sensors were placed strategically to obtain values from key points such as the hallux, central and lateral midfoot, central and lateral forefoot, the first metatarsal and the heel. The sensors were also suitable for stimuli between 25 to 250 kPa. Although this may be ideal to monitor walking, it may not be suitable for other physical activities where parts of the foot may exert higher pressures. Furthermore, this device requires the users to calibrate the sensors prior to use due to the sensor performance degrading with time. This device was validated using three subjects, all of whom were female. However, two subjects were 89 years old, and one was 22 years old. An advantage of this sample is that the system was shown to be usable and accurate for both young and older individuals. However, including male subjects to account for foot morphology differences and using a larger sample size, would allow for the device’s usability to be generalized to the general population. Another merit of this device is its long battery life, capable of taking measurements for 20 h continuously. Although such a device appears to overcome several challenges such as the need for creating a thin sensor system that has a prolonged run-time, the cost of this device exceeded USD 1000. 

A sensorized insole prototype for pressure distribution measurements was developed in [77]. A large number (336) of tactile capacitive sensors in a mesh was used in this work. Each insole had 336 capacitive sensors, arranged among 28 triangular regions and covered most of the insole. Of the 336 sensors, 56 sensed temperature, and 280 sensed pressures. The capacitive transduction electronic portion is covered by a deformable dielectric material, on top of which is a conductive lycra fabric. This increase in spatial resolution can allow for an improved understanding of the PPD under the entire foot. However, this prototype did not support wireless transmission as yet. This device also includes a variety of components, such as the electronics, soft fabric for user comfort and plastic support for protection. However, the authors indicated that the comfort can be further improved, using stretchable electronics or adding softer supports for the electronic portion. 

Insoles based on PPD that are customizable to the user were created in [78]. Although this system does not measure PPD itself, it makes use of predetermined plantar pressure measurements to create a highly customized insole. This insole is created using 3D printing to obtain a customized shape for the insole, in addition to using a variety of infilling densities specific to the user. In order to create such an insole, PPDs were first obtained using a piezoelectric sensor array. Based on this information and processing of the data, the densities of the infill were determined, and this information was collectively used to create the 3D-printed model. This work highlights the feasibility of creating custom orthotics that are precisely tailored to the wearer. However, this technology can be taken further to include in-built pressure sensors within the custom insole, particularly for individuals who would use monitoring devices for extended durations and over the long-term. The elderly population would greatly benefit from a highly customized insole which also measures their plantar pressure. Individuals in this age group are at an increased risk of falling, and thus, it is important to monitor PPD and gait characteristics in order to predict a person’s risk of falling.

**Figure 4:**
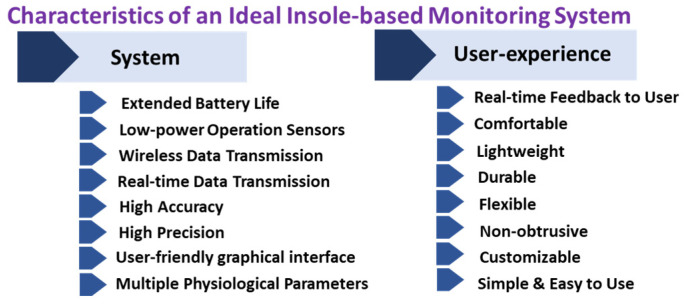
Expected characteristics of an insole-based monitoring system.

The aforementioned insole-based systems [69,71,72,73,74,75,76,77,78] have varying merits and limitations. Developing a device with several beneficial characteristics is ideal, but difficult. This is because attaining some desired characteristics often results in compromises of others. For example, increasing the number of sensors to obtain more detailed measurements also increases the power usage, which in turn reduces the battery life. Figure 4 lists some hardware and software characteristics of an ideal insole-based health monitoring system. Future insole-based systems should attempt to incorporate these ideal characteristics as appropriate for the intended application. A summary of key points pertaining to the aforementioned systems [69,71,72,73,74,75,76,77] is presented in Table 1.

#### 3.1.2. Triboelectric Sensors for PPD and Related Applications

Recent investigations on self-powered devices have allowed for advances in smart insole-based and related systems. In particular, triboelectric nanogenerators (TENG) were used to allow for devices to generate power for sensors. Triboelectric sensors were created for the purpose of energy harvesting [79] and have since been used for a variety of applications [80]. A few examples of these diverse applications include detection and monitoring of airflow, vibration, pressure, gait, temperature and pulse [79,81]. Regarding insole-related wearable applications, triboelectric sensors have been incorporated on insoles, embedded in the sole, engineered into the sole, integrated into the sole as well as beneath the sole [82] to generate electricity.

At the surface of two materials that are contact with one another, but which differ in their ability to attract electrons, opposite charges are generated. Once these two materials are separated or displaced, a potential is created, which leads to the flow of electrons [79]. The result of the force applied is the generation of electricity, resulting in self-powering capabilities. TENG can be divided into four main types which are “contact-separation”, “linear-sliding”, “single-electrode” and “freestanding triboelectric-layer” [79].

In “contact separation”, the physical contact between the two materials is removed due to an external force, resulting in a potential created in the interfacial gap of the materials. In “linear sliding”, sliding causes the contact areas of the two surfaces to be altered. In this case, the sliding may disturb the electrostatic stability and create a potential. The “single-electrode” mode has a freely movable dielectric portion that is not physically connected to an electric component. The transfer of charge occurs as the dielectric moves to and from a stationary electrode. The “freestanding triboelectric layer” mode has two electrodes that are ground-free. When a dielectric moves between both electrodes, induction results in a charge distribution that is not symmetrical [79]. In addition to self-powering capabilities, other characteristics of TENG include its low cost, light weight and stability [79], which make this technology particularly useful for smart insole-based applications. Several researched systems which have incorporated TENG are summarized in Table 2 below.

#### 3.1.3. Gait and Activity Monitoring

In recent years, insole-based human gait and activity monitoring systems are gaining prominence because of their ability to combine different wearable sensors such as IMUs, pressure sensors and temperature sensors, and to unobtrusively measure important gait parameters [90,91,92,93,94,95,96]. Although, the sensor types used to develop these insole-based systems are quite similar (except the number and placement of the sensors), the main differences reside in sensor fusion and data analysis algorithms used for human gait feature identification, activity recognition as well as fall detection and prevention. In this section, several developed insole-based systems for gait and activity monitoring are presented.

Several researchers have developed insole-based gait analysis systems integrating pressure and inertial motion sensors. “FreeWalker” was such a gait analysis tool [90] that comprised of eight pressure sensors and two IMUs to measure under-foot pressure distribution and to detect motion sequences during gait. An onboard SD card and integrated wireless data transmission enabled this system to have continuous monitoring and real-time gait data analysis. Another smart insole was proposed in [91] for ambulatory gait assessment. This system consisted of a sensor layer with 32 discrete pressure sensing points (piezo sensors) and a circuit layer with the processing unit as well as an IMU and a temperature sensor. Similar types of smart insoles were also reported in [92,93,94]. In [92], the researchers used an array of electronic textile-based pressure sensors. All these systems were able to measure the plantar pressure and motion characteristics of feet during walking. In [93,94], the developed insole systems were validated against the gold standard GaitRite (CIR Systems Inc. Clifton, NJ, USA) and showed adequate accuracy while measuring important spatiotemporal gait parameters such as stride time, stance time, swing time, step time, initial double support time and terminal double support time. GaitRite is an electronic walkway with built-in pressure sensors, and it can provide standard spatiotemporal gait parameters using its software application.

Insole-based gait monitoring is also effective for gait balance and dynamic stability estimation by measuring the center of mass (COM) and margin of stability (MOS) of the human body during walking [95,96]. In [95], the researchers used an existing Pedar insole and a newly-developed piezoresistive smart insole to estimate the center of pressure (COP) during gait and compared the results with a force plate. For both insoles, the accuracy of estimated COP was lower in the mediolateral direction in comparison with the anteroposterior direction. In order to estimate accurate COM and MOS during gait, Xsens Technologies developed force- and motion-measuring shoes named “ForceShoe” [97]. This shoe consists of two IMUs and two 3D force and moment (F&M) sensors to measure 3D forces and moments as well as foot positions during walking. However, this shoe is large and bulky. Therefore, as an alternative, researchers in [96] evaluated 1D plantar pressure sensing and developed participant-specific linear regression models to estimate 3D F&M. Then they combined the estimated 3D F&M with foot positions (measured using IMUs and an ultrasound range estimator) to compute the COM and MOS during gait. Overall, the study showed satisfactory results for straight walking.

A human motion capture system was reported in [98] based on multiple IMUs and smart shoes. The IMUs placed on different body segments were used to estimate the posture of the whole body, while the smart shoes with pneumatic pressure sensors were used to detect the phase of the motion during gait and other activities. The smart shoes were continuously measuring the ground reaction forces to update the reference point of motion. A frequency-adaptive sensor fusion technique and a kinematic model were applied in this system to improve the precision of the measurements and to track real-time whole-body motion.

Besides estimating important gait parameters, smart insole-based gait analysis is also used for gait classification and user identification [99,100,101,102,103,104,105]. Various classification algorithms have been proposed in different literatures to classify the gait data collected from smart insoles. For example, the support vector machine (SVM) algorithm was applied in [99] to distinguish three types of walking. In [100,101], principal component analysis (PCA) was used to analyze the gait data and identify abnormal gait patterns. Researchers in [102] used a commercial “FootLogger” smart insole to collect data from seven different types of gait such as normal walking, fast walking, running, stair ascending, stair descending, hill climbing, and hill descending. They applied null-space linear discriminant analysis (NLDA) to classify the data and obtained good classification performance. They also observed that the classification performance becomes better with a larger number of steps in the sample. Similar seven gait types were also classified in [103] using Deep Convolution Neural Network (DCNN) and presented high accuracy of more than 90%. Another automatic smart insole-based gait recognition method named “BoostSole” was proposed in [105], and an adaptive multi-boost classification algorithm was employed to classify three different gait patterns: normal walk, shuffle walk and toe walking with a very high accuracy of 97%.

Finally, there are several other studies where researchers proposed different smart insole-based gait analyses to evaluate human gait patterns in different health conditions and rehabilitation processes. For instance, the FeetMe Monitor insole was used in [106] to evaluate after-stroke gait of 29 subjects (over 6 months since stroke) and showed high accuracy and reliability (Intra-class Correlation Coefficient—ICC: 0.73–0.98) while measuring gait velocity, stride length, cadence, and stance time in chronic hemiparesis. In [107], the authors introduced an insole-based gait analysis system (PediaSole) for toddlers and children with neurodevelopmental disorder (NDD) and/or autism spectrum disorders (ASD). Children with NDD and/or ASD usually suffer from motor impairments and precise gait analysis can play a vital role in early diagnosis and effective treatment strategies. Therefore, PediaSole was used to measure gait parameters of child subjects with these disorders and showed high accuracy (mean absolute errors—MAEs were below 5.2% for all gait parameters) in comparison with the gold-standard equipment. This study also found high correlations between three measured gait parameters (normalized stride velocity, stride time, and stance percentage) and a well-established clinical measure of motor function (GMFM-88—E) which indicates the potential of the insole-based system for similar clinical applications. In addition, a few other studies used smart insole-based gait analysis for monitoring post-surgery effects [108] and for the identification of fall hazards [109]. Thus, smart insole-based gait monitoring systems coupled with efficient processing and analysis techniques can be more widely used for remote health monitoring, rehabilitation processes as well as different clinical applications such as detection of diabetic neuropathy and prevention of ulceration in the insensate foot [110]. A summary of the aforementioned insole-based gait and activity monitoring systems is presented in Table 3.

#### 3.1.4. Other Physiological Parameters

##### Heart Rate Detection

In [111], the estimation of heart rate using photoplethysmography sensors worn at the foot was investigated. This system was particularly used to estimate heart rate during fast biking exercise. Heart rate measured by wearable sensors was typically done using sensors placed at the wrist. However, this system is an alternative approach which also provides the potential for energy harvesting, a benefit unique to this type of design. However, the fast motion during fast bike exercise also causes the heart rate measurements to be more difficult to obtain. Eight participants were used to verify the heart rate determined by the system. This system was capable of estimating average heart rate with a 9-bpm error. Although the results indicated that more work is required for such a device to be used for accurate clinical measurements, this unique concept of utilizing foot-worn sensors can be applied to insole-based systems to measure a variety of physiological parameters. The noise may be reduced by using the device in activities requiring less motion (such as walking instead of running). However, this would compromise the energy harvesting benefit of these devices. Improving the algorithms used to remove motion artifacts can also help improve such a device for practical purposes.

Heart rate was also aimed to be detected in [112], but by using a weighing scale. As opposed to the traditional method of using electrodes and having current passing through both feet, this work provided an alternative where only one foot is required to detect heart rate using impedance plethysmography (IPG). It is indicated that this system is particularly beneficial for individuals who may have an amputated limb, pacemaker users, electronic implant users and pregnant women. This system allows for an individual to stand on a weighing scale for the measurements to be obtained from a single foot (as opposed to traditionally using both feet). In particular, the pulsatile component from the measured plantar bioimpedance is used to detect the heart rate. It is also reported that the recorded signal contains information regarding respiratory rate. The ability of such a system to provide a variety of physiological parameters may be useful in developing future devices for monitoring vital signs, or monitoring and reporting several physiological parameters, including heart rate and respiratory rate, through a single system. 

Ballistocardiography (BCG) was also used to determine heart rate in an insole-based setting. An insole with piezoelectric films to measure heart rate was developed in [16]. In the initial stages, the signals obtained were weak. However, when the participants exercised, the signals were enhanced due the greater cardiac output as a result of exercising. Ballistocardiography is a possible option to consider for measuring heart rate in an insole-based format, but may be limited to certain situations such as measuring heart rate during or after physical activities.

A wearable system for the foot which is able to detect changes in muscle activity in addition to detecting the pedal pulse at the dorsal aspect of the foot was developed in [113]. The device, called “FeetBeat”, has sensors at the tongue of the shoe to detect the pulse from the dorsal pedal artery. This artery may be ideal to target for measuring pedal pulse if a device such as a shoe were being designed. However, it may not be feasible to target the dorsal pedal artery in an insole-based system taking measurements at the plantar aspect of the foot (the sole).

In [17], heart rate was determined from the sole of the foot by obtaining signals from the lateral plantar artery at the plantar aspect of the foot. A major challenge with obtaining heart rate measurements from the sole of the foot is that fat pads exist for the protection of the foot and for distributing an individual’s weight. However, these fat pads are additional layers of biological tissue which increase the distance between the target artery and the proposed location of the sensor. The fat pads are particularly prominent under the metatarsal heads of the foot and the heel. However, in [17] it was found that the midfoot was the optimal location to obtain heart rate measurement using PPG sensors. In fact, the lateral plantar artery can possibly be targeted at the midfoot, particularly at the lateral midfoot at the plantar arch.

##### Muscle Activity Detection

A system which measures lower-leg muscle activity in a sock-type wearable sensor system was developed in [19]. This system uses distal EMG signals to determine muscle activity. Electrodes placed at the surface of the skin are able to detect the biopotential signals which result from muscle activations. Some benefits of using electrodes in a sock include the tightness of the sock fabric, and preventing the electrodes from sliding out of their appropriate placements. In this design, electrodes were placed at the lower leg (at the calf and near the ankle), as opposed to the foot. The conductive fabric electrodes make contact with the skin on one side and with the sock fabric on the other side. The muscles’ activities measured in this design include the tibialis, gastrocnemius, soleus and peroneus. This is accomplished using six sensors positioned on each leg. To gain more holistic measurements of foot plantar pressure and lower limb muscle activation, an insole-based system with a wearable sock that extends up to the lower leg can be useful to obtain measurements of muscle activity, in addition to parameters such as heart rate, temperature, plantar pressure and gait characteristics.

##### Temperature Measurement

A flexible smart insole based on PDMS/CNT (polydimethylsiloxane/carbon nanotube) was developed in [114] that is capable of measuring and mapping temperature and pressure of the foot. This system uses pressure sensors and thermistors placed the hallux, 3rd toe, forefoot, lateral midfoot and heel. Twelve pressure sensors and four thermistors were placed in the aforementioned regions. Electrodes were printed on a highly compliant flexible substrate. Such compliance is ideal for use in insole-based systems as the insole can adjust its shape along with the movement of the foot, without damaging the insole while maintaining the capability to record measurements. Using a scalable mesh-molding process, the pressure sensing microstructures were fabricated. It is indicated that using printed side-by-side electrodes allows for sensor arrays to easily be created, in addition to having an increased sensitivity and greater working range than top-bottom electrodes. In fact, the working range of this sensor system is from 7.4 Pa to 1 million Pa. The change in pressure results in a resistance change between the two electrodes because the conductive microstructures change. Insole-based sensor systems that are highly compliant and can withstand a broad range of applied pressure can be highly beneficial for a variety of activities where the foot shape and applied pressure may vary greatly. Furthermore, being able to measure temperature along with pressure can serve two purposes. First, for sensor systems which work within a specific temperature range, temperature sensors can ensure the readings obtained are reliable based on the temperature at which those pressure measurements were obtained. Second, temperature at the foot may also be a physiological parameter of interest to be periodically measured, such as for individuals who may suffer from Grierson-Gopalan syndrome (also known as “Burning Feet syndrome”), or if an individual may suffer from Raynaud’s phenomenon (where toes and fingers drastically decrease in temperature) as well as for the detection of diabetic foot ulcer risk, which is associated with an increase in temperature at specific points at the foot sole [18]. Early detection of the onset of such phenomena may help reduce the adverse physiological effects one experiences, by taking measures to reduce the effects based on early readings of temperature changes from such an insole-based system. 

**Figure 5:**
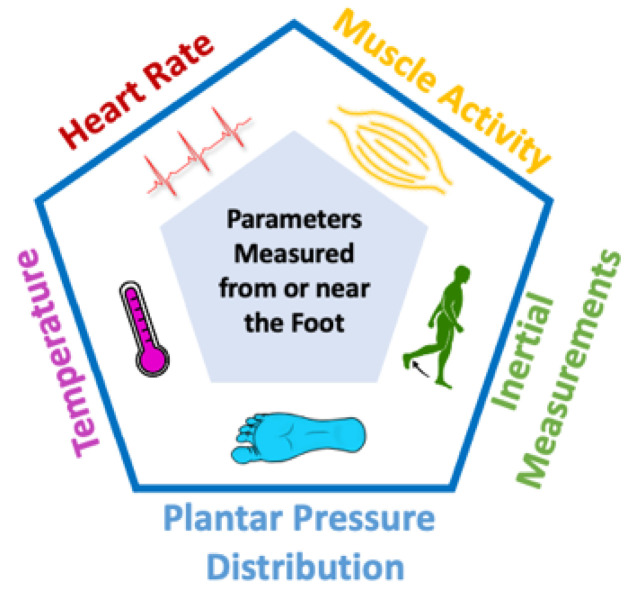
Parameters which have been measured at or near the foot.

In summary, several insole-based or related systems were reported in the literature that can measure a number of parameters from the foot or near the foot. The parameters for gait and health monitoring which were investigated include PPD, inertial measurements, muscle activity, heart rate and temperature, as shown in Table 4 and Figure 5.

### 3.2. Commercially Available Systems

Certain insole-based sensor systems, which measure parameters such as foot pressure distribution, are currently commercially available. These “smart insole” systems typically aim to be a supplementary device used for activity monitoring, particularly for those who engage in athletic activities. 

An insole system developed by Salted [115] measures foot pressure, gait patterns and bodyweight distribution. The insole contains four sensors and has benefits of being trimmable (to be customized to an individual’s particular foot or shoe shape) and is waterproof. This device is typically used for pressure analysis when golfing, and provides wireless data transmission over Bluetooth to a smart device (such as a phone) in real time. The Salted insole is also capable of providing vibratory feedback through an individual’s phone to indicate when the individual achieves their desired weight distribution during the golf swing, based on the pressure data obtained [116]. Features such as trimming the insole and using a waterproof material or coating are beneficial to improve all day use and account for foot morphology differences.

The “UniverSole” by IPPINKA [117] is a commercially available insole with pressure sensors. IPPINKA pairs the insole with a mobile or computer app which can show images of pressure mapping to indicate the pressure distribution across the feet, in addition to displaying what percent of body weight each foot is bearing. The mobile app also provides notifications indicative of which foot is receiving more weight and provides progress updates regarding achieving an individual’s daily step count goal. Graphical user interfaces such as the one used with the “UniverSole” can provide an easy way for users to monitor their fitness goals and foot pressure distribution, while obtaining feedback in an easy-to-understand manner.

Another insole-based system was developed by Arion [118]. This company created an insole which contains eight pressure sensors distributed throughout each sole. These insoles are described as being thin and flexible, with a thickness of only 2 mm. This insole is also available in four sizes, which can accommodate several, but not all potential users. This insole system works with a “sensor packed footpod” which is a device that connects to the wired portion of the insole. This “pod” is able to be attached to the side of an individual’s shoe and has a gyroscope, accelerometer, global positioning system (GPS) and Bluetooth for wireless data transmission. An additional benefit of this “pod” is the LED lights which change to various colors to indicate when measurements are being taken, if it is improperly connected, or the device is charging properly, etc. It is described as being small and lightweight, with seven hours of battery life. This battery run-time is ideal for the intended application of this device, which is geared towards tracking activity during running. 

The Arion device maps the run path, pace and duration of an individual’s run. It also identifies which part of the foot strikes the ground first, in addition to an individual’s step length, cadence, foot contact time (indicating the gait cycle’s stance phase duration), and time spent with the foot in the air (indicating the swing phase duration). The device is also able to provide information regarding an individual’s balance based on weight distribution in both feet, and stability of the foot. The parameters measured by Arion’s device can be very useful for applications in a variety of populations. One such population that would benefit from the measured variables include older adults, as the system can be used for gait characteristic monitoring and for improvements in health. Although the Arion device is not intended for all-day wear, this device measures a multitude of variables, which would particularly be beneficial for monitoring activity and gait characteristics of elderly individuals. For instance, the inertial measurement units can be used to detect falls or slips, and information regarding the frequency of falls or severity of falls can be beneficial to clinicians. Gait characteristics can also be used to predict fall risk, which is also an indicator of overall well-being [31]. Activity monitoring and relaying the acquired information in an app-based format can also encourage older adults to be more active and reach certain fitness goals (such as engaging in a certain duration of exercise or achieving a predetermined number of steps). Embedding GPS trackers in an insole-based system may also have additional benefits. One use of a GPS is to modify walking routes based on previous taken routes, in order to enrich sensory stimuli of the individual. Exposing one to new sensory stimuli, such as nature during outdoor walks, may also improve mental health. Being exposed to varying sensory stimuli can help improve the quality of life of older adults in addition to having fulfilling experiences outside of their home. Furthermore, several older adults with dementia are under constant supervision by caregivers [119]. However, tracking an individual using a GPS, such as during their outdoor walks, can allow for older adults to have more independence in their daily activities, as well as reduce anxiety and worry for caregivers. However, the privacy concerns of GPS tracking would have to be mitigated if data is accessible by individuals other than the user of the insole. 

**Figure 6:**
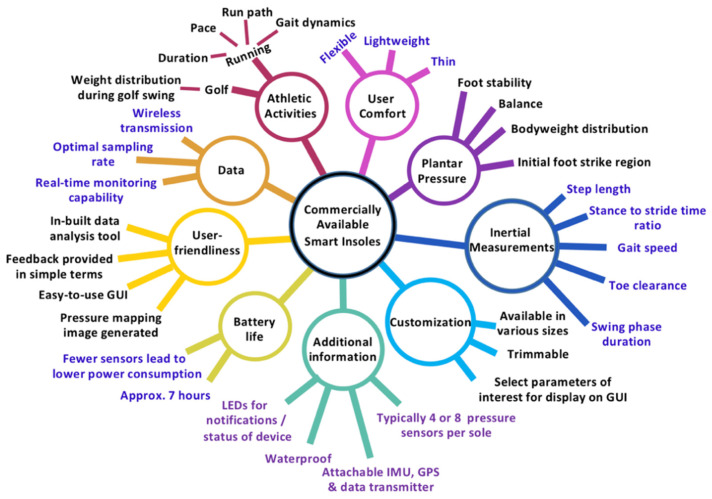
Characteristics of several existing commercial “smart insole” pressure measurement systems.

Commercially available smart insoles are typically designed for athletic activities to monitor and improve performance. Although such systems provide useful information regarding an individual’s plantar pressure and characteristics related to gait, a greater number of sensors can be used to improve the pressure data obtained. However, several characteristics of the currently available smart insoles are admirable and highly beneficial, and future insole-based health monitoring devices should incorporate such characteristics when and where possible. Such characteristics found in commercially available smart insoles are outlined in Figure 6.

## 4. Design Aspects and Considerations

### 4.1. Sensor Selection

Insole-based sensors systems can enable an unobtrusive means of health monitoring by incorporating a variety of sensors. For example, plantar pressure can be measured by integrating a set of resistive, capacitive, piezo-resistive, or piezoelectric pressure sensors in the insole [41]. Other sensors that were also used in the literature for pressure detection include optical sensors [75] and MEMS (micro-electromechanical systems) sensors [120]. These sensors detect changes in pressure and convert it to an electrical value corresponding to the applied force. IMUs can further be integrated into the insole that can potentially enable analysis and assessment of gait in a wearable platform.

However, selection of appropriate sensors is critical in order to ensure the accuracy and clinical relevance of the measured signals. For example, pressure values vary widely across the foot plantar from about 157 kPa in the third metatarsal region to as high as 631 kPa in the entire forefoot region [121] and can reach up to 1900 kPa over the entire foot [41]. Therefore, the number, sensing area, and location of the sensors as well as the intended resolution of plantar pressure distribution are some key factors that must be taken into account while selecting the sensors [41].

In addition, the ambient temperature inside the shoe can vary widely [122], which can in turn affect the performance of the system. It was reported that the temperature inside the shoe can increase by 6 °C in 90 min when worn, even without any physical activity [122] and by about 8 °C in 45 min when walked at a speed of 0.7 ms^−1^. Since the temperature within the shoe can change significantly within a short amount of time with little to no physical activity by the user, pressure sensors embedded in the insole must have a low temperature sensitivity, particularly in the range of 20 to 37 °C [123]

On the other hand, IMUs are robust enough to withstand a wide range of temperature. Therefore, change in the ambient temperature is not a key concern for IMU selection. The key selection criteria of the IMUs for gait monitoring and analysis purposes are their dynamic range (DR), resolution and sampling rate. Higher DR, resolution and sampling rate of the IMUs enable measurements of gait dynamics with higher precision; but the penalty is reduced battery life and large storage requirement of the system [124]. Therefore, the selection criteria of IMUs may vary based on the application. For a system to capture gait dynamics during the day-to-day activities such as walking, and running, an accelerometer with a DR of ±8 g [124,125] and a gyroscope with a DR of ±2000 deg/s are generally adequate [125,126].

### 4.2. Data Acquisition Rate

When acquiring a variety of signals, it is important to consider the optimal frequency or rate for collecting the signals. This data acquisition rate needs to be sufficiently high for an accurate, reliable and clinically relevant representation of bio-signals, while minimizing the power consumption for the portable systems to facilitate extended battery life.

In the case of a smart insole device, the main purpose is to obtain data related to foot health, activity and gait characteristics. Therefore, it is critical for pressure and inertial measurements to be obtained consistently. By ensuring signals are collected continuously at an adequate rate while the insole is in use, a more holistic view of an individual’s PPD and gait characteristics can be obtained from the larger volume of data acquired over longer periods of time. For the purposes of PPD measurements, a sampling rate of 50 Hz should suffice while walking [127]. However, a sampling rate of at least 200 Hz is required during running [41]. Similarly, while a sampling rate of 50–100 Hz is sufficient for IMUs to measure gait movement (acceleration and angular velocity) during different pace of walking [2,128], a higher sampling rate (>250 Hz) is required to capture the detailed aspects of gait patterns during running [129,130].

In addition to PPD and gait monitoring, a smart insole can incorporate sensors for monitoring pulse rate [17] and temperature [18]. Daily measurement of pulse rate can provide insight regarding an individual’s cardiovascular and overall health. The smart insole can facilitate monitoring pulse rate (PR) and pulse rate variability (PRV) continuously for longer periods of time or at rest, or before and after exercise, as demanded by the user. Although a lower sampling frequency is acceptable for measuring the PR, a sampling frequency of at least 100 Hz is required for time-domain analysis of PRV [131]. Temperature sensors used in such an insole-based system serve multiple functions. For the purpose of detecting diabetic foot ulcers, it was determined that monitoring foot temperature once a day can be an effective preventative measure for individuals who have a higher risk of foot pathologies related to diabetes [132]. To ensure sensors are able to accurately operate within the specified temperature range, temperature measurements should be taken more frequently to account for the temperature changes of the foot within the insole or footwear environment.

### 4.3. Data Analysis: Sensor Fusion, Prediction, and Decision Making

A system consisting of different types of sensors allows for collecting multimodal information and performing comprehensive analysis. The information from multiple sensors embedded in an insole can be combined or fused to perform more complex analysis, overcome respective limitations of each sensor and achieve better results with improved resolution, high reliability, larger spatial–temporal coverage, reduced ambiguity, and greater precision in measurements [133,134]. In addition, by combining the information from different sensors, the context and perspective of the circumstances can be assessed, thus facilitating a better understanding and assessment of the health and wellbeing of the users.

**Figure 7:**
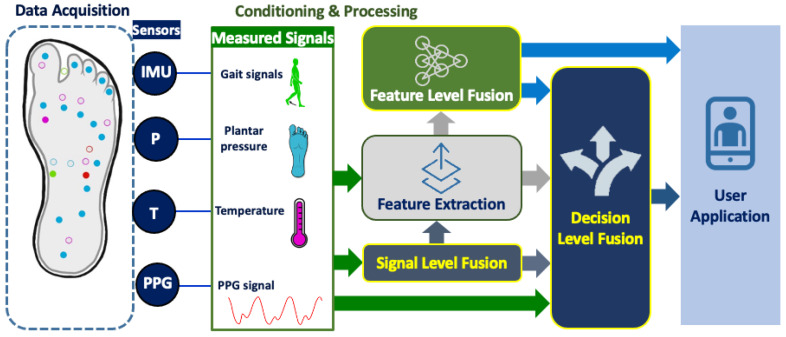
Sensor fusion in a multimodal insole-based system.

The sensor fusion can be performed at three levels: signal level [135,136], feature level [103,104] and decision level [103,104]. In signal level fusion, preprocessed signals from different sensors are combined using several techniques such as weighted averages, complementary filter, Kalman filter and particle filtering to obtain usable features for further analysis. At the feature level, sensor fusion is performed by combining the feature vectors extracted from the sensors’ data. This fusion level can be used for dimensionality reduction of feature space, and it results in a higher-level feature representation of the sensor data [137]. Principal component analysis (PCA), k-nearest neighbor (k-NN), decision trees (DT), support vector machines (SVM), Gaussian mixture model (GMM), k-means, artificial neural network (ANN) and deep learning are the most commonly used methods to perform sensor fusion at the feature level. Decision level is the highest level of fusion that is used for recognition and decision-making purposes. The input to this level can be the outputs of the previous two levels and/or the raw data from the sensors. Probabilistic techniques (e.g., Bayesian inference) are generally used at this level due to having high level of uncertainty [134]. However, other algorithms such as fuzzy logic, weighted decision, artificial intelligence, and genetic algorithms are also frequently used for decision-making. An overall process flow of sensor fusion technique is presented in Figure 7.

Several researchers have proposed different multimodal insole-based monitoring systems and used machine-learning approaches to fuse multi-modal data/information in an attempt to evaluate human gait [103]. In [104], a pressure sensor array, and an array of accelerometers and gyroscopes were used to develop a smart insole. A deep convolution neural network (DCNN) was employed to classify different gait types. There, they extracted gait features from individual sensor arrays and then generated a fully connected network by combining the features. The team was able to distinguish seven different types of gait (including walking, running, stair climbing, etc.) with an accuracy of over 90%. The team also indicated that such a system can be used for earlier diagnoses of diseases, such as for knee osteoarthritis and neurological disorders such as Parkinson’s disease. In [124], the researchers used the null-space linear discriminant analysis (NLDA) algorithm to extract single-modal features from the gait data obtained from an accelerometer and pressure sensors embedded in an insole for gait pattern identification. The information from three sensing modalities (acceleration, pressure, and a combination of both) were used separately and their performances were compared. The authors reported achieving a gait identification accuracy of more than 95% with the multi-modal features. A textile-based insole sensor system was proposed in [136] to measure planter pressure and wetness during walking, with pressure and wetness sensors. The researchers used this multi-modal design for long-term monitoring and analyzing of dynamic skin interface environments to facilitate highly-customized orthotic/orthopedic solutions. Gait type classification using pressure and gyroscope sensors was also used to identify walking paths (curved or straight walking) [138]. Another study identified gait cycle events such as swing and heel-strike based on ankle-mounted IMU data from treadmill usage [139]. 

Machine learning techniques are now widely used in recognition and classification processes, as well as for identifying various physical and movement disorders during daily activities [140,141,142,143,144,145,146,147,148,149,150,151,152,153,154,155]. Machine learning is a method of automation that includes different mathematical functions and algorithms, and it has the capability to learn and generate estimations based on the provided data [156]. A machine learning technique usually works by building a data-driven mathematical model to make estimations and produce decisions based on sample inputs rather than strictly following static program instructions. Machine learning techniques can be divided into three types: supervised learning, unsupervised learning, and reinforcement learning [157]. In supervised learning algorithms, the feature vectors include the labelled data in order to explain better relationship between input feature vectors and their corresponding labels. The commonly used supervised algorithms in activity and gait analysis are support vector machine (SVM), random forest (RF), neural network (NN), k-nearest neighbor (kNN), decision trees (DTs), hidden Markov model (HMM), and ensemble learning. In contrast, in unsupervised learning methods, no labels are provided. The algorithm itself formulates the relationship between various inputs to estimate an output. These techniques are mostly used when the correlation between different observations is unknown, and they are capable of identifying distinct patterns for particular disorders. Therefore, unsupervised learning techniques are often used for clustering of abnormalities and detecting asymmetries in the gait data. A reinforcement learning model is capable of considering and interpreting its environment, generating actions (reward or penalty) and learning through trial and error. Due to its ability to recognize data heterogeneity and simplify according to a particular condition, reinforcement learning and deep neural networks (DNNs) are now very popular in rehabilitation studies and recovery devices such as walking assistive devices and exoskeletons. Studies related to activity and gait analysis using advanced machine learning techniques are summarized in Table 5.

### 4.4. Information Dissemination, Interaction and Feedback

Another important aspect of an insole-based health monitoring system is the handling of the data acquired. For instance, the data needs to be presented in a simple and easy to understand manner to the user; whereas, the detailed data may be of interest to medical professionals such as the user’s physician for health assessment purposes. The user may also want to receive feedback about their health and make interaction with the system through a user-friendly graphical user interface (GUI). An effective method of sharing data acquired from a smart insole was proposed in [158]. There, they exploited an internet of things (IoT) framework to collect, monitor and distribute the data to the authorized individuals, along with using a smart insole. Such an IoT system can enable tele-monitoring of health, thus improving the efficiency of health care by allowing physicians to have remote access to data reflective of physiological parameters.

Another system that allows for gait patterns based on accelerometer data to be analyzed and displayed is iGAIT [159]. As a software developed in MATLAB, iGAIT allows for extracting gait parameters that include spatio-temporal, spectral, regularity and symmetry features. This system aims to be interactive, as the extracted features are also displayed to the user, in addition to storing the information for later use. The software also enables users to alter how their gait is analyzed based on their specific needs.

A mobile companion robot that can be used in conjunction with an insole-based sensor system was developed in [160]. This system used IMUs and force sensors in the in-shoe based system, and the red–blue–green-depth (RBG-D) sensors on the robot (Kinect v2). The robot serves several roles, including monitoring an individual’s gait and facilitating socialization. The latter is done by having the robot act as a companion for individuals such as older adults who may be socially isolated, and enabling teleconferencing calls for socialization in a virtual manner. These mobile robots were developed to guide walking sessions, and to monitor gait and provide corrective warnings and feedback to the user. The robot exploited the data from the in-shoe system and the sensors on-board the robot to interact with the older adults and to navigate. The insole-based system has a faster sampling rate than the robot system (500 Hz and 30 Hz, respectively). The robot’s Kinect system can also obtain information such as the step length without drift. The insole system on the other hand is able to provide detailed information regarding gait characteristics such as foot pressure. The synergistic effect obtained from using information from both the in-shoe system and the robot allowed for more detailed information to be obtained. This results in greater estimation accuracy of gait parameters such as foot clearance, stride velocity and stride length, compared to using only one of the systems. Although this system is able to provide useful measurements and data, there still exist certain limitations. For instance, the distance between the user and the robot was reported to affect the accuracy of the data measured by the robot sensors. The robot also used markers to estimate gait events that may not be accurately measured in environments that differ in lighting, or when the subject’s distance from the robot increases and may not be practical in day-to-day use. Nevertheless, this robot-insole combination can potentially increase the independence of older adults as their reliance on caregivers decreases, since the system takes over the task of constant monitoring. Furthermore, the use of artificial intelligence (AI) can enable the robot to make intelligent decisions about the overall wellbeing and safety of the user.

## 5. Research Challenges and Future Perspectives

### 5.1. Research Challenges

**Figure 8:**
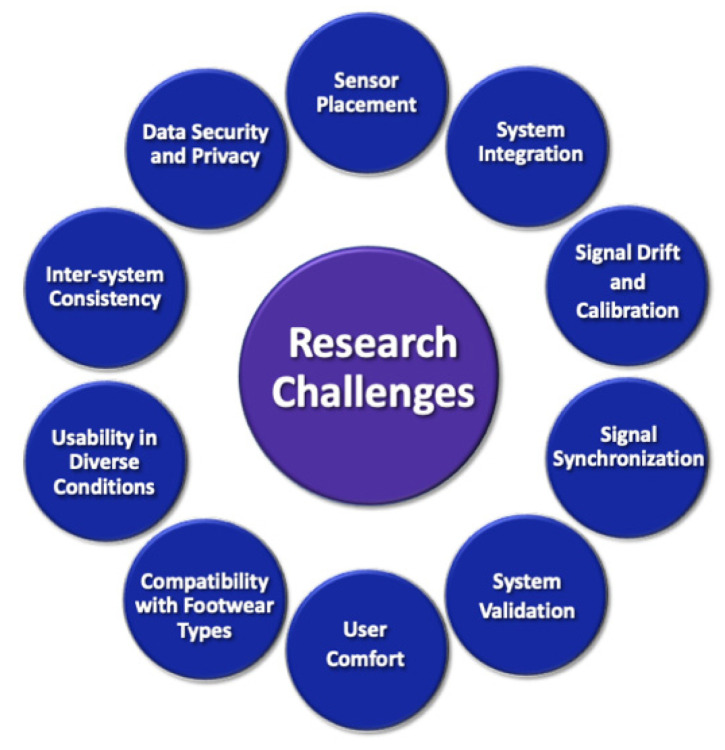
Research challenges associated with an insole-based health monitoring system.

There are several challenges associated with the development and use of insole-based devices to improve their performance and usability. Ten challenges are summarized in Figure 8 and are described below.

a. Sensor Placement

Unlike force platforms which have an array of sensors that allow them to measure pressure across the entire foot, the insoles use discrete sensors at particular regions of the foot. It was observed that the placement and size of the sensors can affect the measurements accuracy [41]. It was indicated in [41] that the sensors should not be placed directly at the area of maximum pressure but rather positioned close to this area. In addition, the surfaces of the sensors must be at least 5 × 5 mm in size, but not too large as they would provide lower values corresponding to the average pressure of the areas of interest. As a result, if sensors are not placed in optimal positions in the insole-based system, accurate measurement of plantar pressure may not be obtained.

b. System Integration

An insole-based system aims to take measurements in the user’s natural setting or during daily activities for an extended period of time. Such systems should have wireless data transmission capability coupled with extended battery life to enable long-term monitoring of gait. Important characteristics of long-term monitoring systems also include having an increased energy efficiency to minimize power consumption. The use of highly efficient batteries, incorporating energy harvesting techniques and using low power components can contribute to achieving low power. The ‘sleep and wake up’ operation of sensors, i.e., sensors being in an inactive stage unless required, can also improve battery life while maintaining the required frequency for measurements [2]. Nevertheless, the integration of several sensors and other electronic components in the insole while ensuring signal integrity and maintaining users’ wearing comfort can be challenging, as more components may cause the device to be heavier or more obtrusive for the user.

c. Signal Error, Drift and Calibration

Insole-based systems have unique challenges in terms of calibration. For example, there may be changes in the shoe environment such as humidity and temperature [75], resulting in measurement drift, thus potentially requiring frequent re-calibrations. In addition, activity-specific and user-specific calibrations may be necessary. Non-linearity of a sensor is another factor that may lead to the need for sensor calibration. For pressure sensors, calibration tables can be used to translate voltage values into the corresponding pressure values to compensate for the non-linearity of the sensor [161]. However, some sensors do not require additional calibration if used within the specified pressure range and within the specified lifetime of the sensor and shoe environment. For instance, OpenGo wearable insole pressure sensors are able to operate up to 400 kPa without the need for re-calibration [162]. Nevertheless, the placement of such a sensor and its sensing area must be carefully considered when used in an insole-based system. When designing insoles for full-day wear and long-term use, frequent calibration may deter users from using such devices. Therefore, it is important to reduce the number of calibrations for better user convenience.

In addition, the orientations of the IMUs in the insole are arbitrary. Therefore, it is critical to estimate and correct the sensor’s orientation to make an accurate assessment of gait. Ideally, a tri-axis accelerometer can be used to obtain the roll and pitch from its position with respect to the gravity vector, whereas the yaw can be obtained by measuring the geomagnetic field with a tri-axis magnetometer. Furthermore, the three-dimensional orientation can ideally be obtained by integrating the angular velocity measured by a tri-axis gyroscope.

However, an accelerometer’s signals are very sensitive to external noise and can get contaminated with vibrations associated with gait anomalies, scar and adipose tissue, that altogether can introduce large errors to the estimated roll and pitch. In addition, the ambient ferromagnetic materials can introduce larger errors to the measurement of the relatively weak geomagnetic field, and thereby in the estimated orientation. Furthermore, orientation estimated through integration of gyroscope data are contaminated with drift.

Typically, measurement from all three sensors, such as accelerometer, gyroscope and magnetometer are used to estimate the device’s orientation. Many researchers exploited Kalman filter-based approaches to estimate the device orientation [163,164,165,166,167]. However, Kalman filter-based approaches generally use complicated and non-linear equations of the process model and the observation model, thus making these methods computationally intensive for real-time applications. Some researchers used linear models of the human musculoskeletal system and the measurement equations to reduce the overall computational cost for real-time systems. However, linearization of the model may result into a system which is sensitive to initial conditions and may have issues associated with convergence and stability, especially at lower sampling rates [68,168].

Regardless, Kalman filter-based methods are widely used in applications such as motion tracking, signal filtering, and sensor fusion due to their high accuracy and speed [169,170]. However, the covariance matrices of the Kalman filter’s process noise and measurement noise need to be determined experimentally. Furthermore, Kalman filter requires inversion of large matrices that increases the computational complexity of the algorithm, and thereby can potentially affect the computing time and the battery life of portable and wearable systems [68,171].

To get around the problems associated with the Kalman filter-based approaches, some researchers used complementary filter (CF)-based estimation approaches [68,128,172,173]. CF-based methods exploit and fuse information from the gyroscope, accelerometer, and/or magnetometer and thus overcome the limitations of each source. CF-based estimation approaches have the advantage over Kalman filter-based methods for reduced computational complexity, requiring only a few tuning parameters and thus making them suitable for real-time systems.

d. Signal Synchronization

One of the major challenges of using multiple types and numbers of sensors in one system is associated with the timing or alignment accuracy of the signals. In a multi-sensor system, it is necessary to synchronize the signals, particularly those varying with time. For signals that do not vary with time—for example, PPD measured at standstill—precise synchronization of the pressure sensor signals may not be critical. However, for signals measured during an activity such as walking and running, where both PPD, as well as gait acceleration and angular velocity change with time, accurate interpretation of the signals may not be possible without precise time-synchronization of the sensors. Synchronization of signals from multiple sources further allows for investigating the associations among different physiological parameters and events, thus enabling a more holistic understanding of an individual’s health. For instance, information such as how an individual’s gait characteristics is associated with an individual’s cardiovascular health may allow for the prediction or indication of more complex physiological conditions based on simple measurements. However, sensors differ in operating principles, output type (analog vs. digital) and output voltage range, response time and sampling frequency, thus often making on-board time-synchronization difficult.

e. System Validation

Most insole-based systems reported in the literature were tested on a small group of individuals of same gender, ethnicity and/or age. This poses a problem as individuals of different genders and ethnicities are known to have different in foot morphologies [174,175]. As a result, a standard placement of sensors may not be suitable for all users of the device. Therefore, ensuring the system’s compatibility with a broader group of individuals can be challenging. Furthermore, force platforms are generally used to compare and validate the plantar pressure measured by the insole-based system [176]. However, the contexts in which the insole can be compared to the force platforms is limited. In particular, force platforms are used in a laboratory setting whereas insole-based systems are designed for use in a variety of natural day-to-day settings. For instance, an individual’s gait characteristics may (intentionally or not) be altered when walking on a treadmill or force platform in a laboratory setting compared to their natural walking patterns. Regarding gait pattern analysis, new systems are typically validated against laboratory-based motion capture systems [177]. Although force platforms and motion capture systems are useful for validation, they may result in measurements that do not reflect an individual’s natural gait, causing validation of the new systems in other activities to be more difficult.

f. User Comfort

As insole-based systems aim to measure PPD during an individual’s daily activities, it is crucial for the sensor to be non-obtrusive to ensure that an individual’s natural gait is not affected by the use of the insole system. This can be achieved by using flexible sensors, comfortable materials for the construction of the insole, lightweight materials and wireless data transmission, in addition to using a battery which is capable of operating during all-day wear. In fact, Bamberg and colleagues indicated that attachments to footwear which are 300 g or less do not significantly impact an individual’s gait [126]. Ensuring the system is in a plug-and-play format also allows for the easy use of the insole-based system, which would in turn encourage the continued use of the product.

g. Compatibility with Footwear Types

An insole-based system is expected to be a ready-to-go wearable device that is compatible with various footwear types. However, achieving ubiquitous compatibility can be a challenge as different footwear types can affect the in-shoe environment (temperature and humidity) and user experience. Due to differences in ventilation capabilities of the materials, the temperature and humidity inside the shoe may vary widely depending on the footwear types and can potentially affect both the performance and calibration requirements of the sensors. Furthermore, the presence of an additional layer of fabric such as socks between the sole of the foot and the sensor system, may affect the accuracy of some parameters, particularly when measuring physiological parameters such as PPD, pulse and temperature.

h. Usability in Diverse Conditions

An insole-based health-monitor is intended for day-to-day use in different activities, environments, weather conditions, temperatures and on varying surfaces. Therefore, the sensors and base materials of the insole should be rugged enough to withstand different conditions and activities. Appropriate ventilation or insulation of the insole (or footwear) should also be considered. In addition, weather conditions such as rain or snow may affect sensors’ performance, so incorporating waterproof or water-resistant aspects into the design can allow for use in such conditions. Surface compliance can also affect the measurements obtained, particularly those of PPD. Furthermore, a stable insole base is critical to pick up ground reaction force consistently and accurately, regardless of the underlying floor compliance type and walking environments.

i. Inter-System Consistency

There exist various insole-based sensor systems reported in the literature and available commercially. However, these systems can vary in measurement capability, range and accuracy due to the different designs. Particularly, the placement and number of pressure sensors on the insole vary widely among the systems that can potentially affect the consistency across devices. For instance, an insole with fifteen sensors located in the optimal positions [70] will provide a drastically different measurement of PPD compared to an insole with only three sensors located at the major foot regions defined as forefoot, midfoot and hindfoot. As a result, clinicians and physicians may find it difficult to compare and assess PPD measurements obtained from different systems with varying placement and number of sensors.

j. Data Security and Privacy

Although wearable wireless systems enhance ease of use and data sharing, they come with risks associated with information security and privacy. As an individual uses the device in varying activities, locations and situations, sensitive personal and medical information are prone to malicious interception. Similarly, as an insole-based system gathers, processes, and transmits a user’s personal and health information, it is important to maintain anonymity and privacy of the collected data. Furthermore, strong encryption is required to ensure data security. However, ensuring secured and reliable communication channels in a low-power platform such as an insole-based system is challenging. The scope of security and ethical requirements of the system has to be clearly defined and specified, and must comply with ethical guidelines recommenced by responsible regulation bodies.

In summary, although certain challenges and limitations currently exist regarding the development of insole-based sensor systems and obtaining PPD measurements, great progress has been made to date, and future research appears to be promising.

### 5.2. Future Perspectives

An ideal insole-based system would incorporate all desired features in terms of wearing comfort, signal integrity, data accuracy, energy efficiency and extended battery life. In addition, current challenges must be taken into account to design a high-performance and efficient insole-based sensor system. However, based on the discussion presented above, four future perspectives regarding the smart-insole are now described.

#### 5.2.1. Multi-Sensor Monitoring System

**Figure 9:**
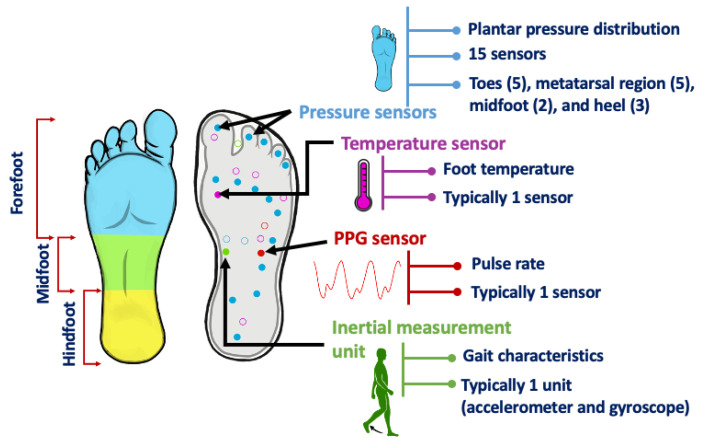
Proposed locations for sensors measuring various parameters in an insole-based monitoring system. Filled shapes indicate ideal sensor locations; hollow circles indicate alternative or additional sensor locations.

The insole can incorporate the capability to measure a variety of health parameters such as plantar pressure, pulse rate, gait pattern and temperature that may potentially enable in-home continuous monitoring of activity, mobility and general wellbeing of the user. One of the key design considerations for a smart-insole is to optimize the placement and number of pressure sensors, as this can affect the accuracy, reliability, and power requirement of the system. In [70], an optimal number of 15 pressure sensors (Figure 9) was recommended for such systems. There, the authors recommended the sensors be placed at the toes, metatarsals, midfoot and heel as these regions of the foot support the majority of the body’s weight and are areas associated with greater variations in pressure [41]. However, depending on the required resolution of the pressure distribution measurements or the application, the number and placements of the pressure sensors may vary. For instance, to determine the relative difference in pressure between the forefoot and hindfoot, three sensors at each of the main foot regions (forefoot, midfoot and hindfoot) can be sufficient. Additional pressure sensors may also allow for detailed insights about foot plantar such as identifying pes cavus or pes planus and deviation in foot arch from normal. However, there is no general recommendation for the placement of IMUs in the insoles, although, IMUs have been placed at the midfoot or on top of the shoe in most prototypes as these locations are generally less obtrusive. Within an insole, a possible placement for this sensor could be at the medial midfoot, as the arch of the foot is typically raised at this location. However, for individuals with pes planus, an alternative placement of the IMU may be necessary, for instance, at the space between the first and second toes [66].

Measurement of the pulse rate from the sole of the foot is difficult due to the presence of fat pads below the arteries of the foot and thus is rarely reported in the literature. However, the midfoot can be an ideal location for a pulse sensor [17], as the lateral plantar artery is close to the skin at this location. In particular, a photoplethysmography (PPG) sensor placed in the insole at a point along the lateral midfoot may potentially enable pulse measurements. Furthermore, embedding temperature sensors in the insole can enable monitoring the temperature of the insole environment to ensure proper functioning of the system in the appropriate range and can also monitor the temperature of foot regions linked to a particular pathology. The temperature sensor placed in Figure 9 is at a location where 52% of diabetic individuals have experienced a foot ulcer [18]. The alternative locations in the figure are other regions that diabetic individuals have experienced foot ulcers. Detecting abnormal temperatures from sensors placed at one or more of these locations may be indicative of diabetic foot ulcer risk.

**Figure 10:**
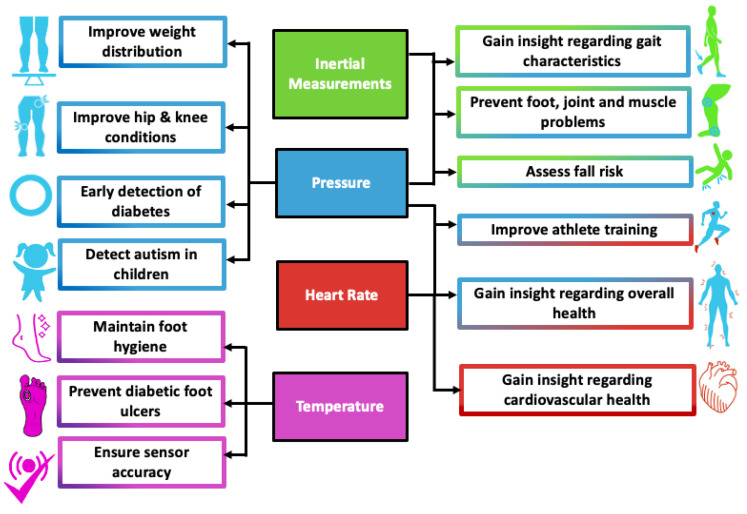
Potential applications of the proposed multi-parameter monitoring insole-based system.

An insole with multiple sensors embedded in it can potentially allow for more efficient monitoring of activity, mobility, and physiological parameters as well as provide physicians and caregivers information that is reflective of an individual’s health during their daily life, as opposed to occasional checkups and laboratory test settings. In addition, different perspectives of an individual’s health can be investigated by measuring, monitoring and combining a variety of parameters as shown in Figure 10.

**Figure 11:**
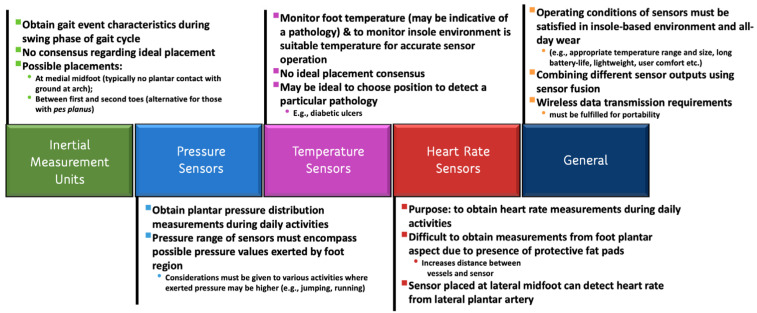
Challenges and considerations regarding the placement and use of various sensors in an insole-based monitoring system.

However, there remain several challenges and considerations (Figure 11) that must be addressed while developing such systems. For instance, in existing publications, sensors such as IMUs, temperature sensors and heart rate sensors were not consistently placed. Considerations must be made to determine the ideal location of sensors to provide the most physiologically relevant information. Additionally, the application that the sensor will be used for (e.g., based on activity level and force impact for pressure sensors) should be taken into account in the system design. Further, barriers to sensor placements such as fat pads at the sole of the foot that may impact PPG measurements, must also be overcome.

#### 5.2.2. Intelligent Feedback System

**Figure 12:**
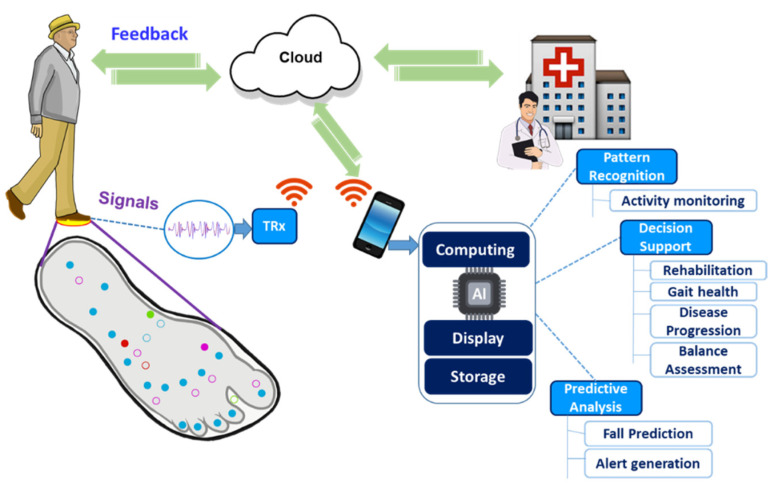
An Intelligent biofeedback system for older adults.

An intelligent biofeedback system (Figure 12) in tandem with a smart insole can potentially be useful in improving the gait and foot health as well as the general wellbeing of older adults, athletes, and individuals recovering from injuries to the lower limbs. Such systems can also be used to assess fall risk by analyzing various gait characteristics including variability in stride length, speed, minimum foot clearance, and double-support stance time [178]. Greater variability in gait is generally indicative of reduced stability and balance that potentially leads to an increased risk for falling. The intelligent system can exploit traditional machine-learning techniques or advanced algorithms such as cognitive dynamic systems [179,180,181] to learn the baseline information about an individual’s gait pattern, activities of daily life, PPD and other physiological parameters. This can allow for trend and predictive analyses of gait, mobility and cardiac health to be performed as well as enable automatic detection of anomalous health patterns such as abnormal gait or cardiovascular abnormalities. The system can monitor an individual’s gait to identify health risks such as an individual’s risk for falling or slipping. Upon the identification of gait anomalies, recommendations can be made to the use to adjust their walking pattern, with the ultimate goal of reducing slips or risk of falls. Such a system can also automatically notify the user and caregivers of anomalies detected, as well as alert if a medical emergency such as a fall was identified. The system can combine the data acquired from sensors with the user’s input to recommend customized and relevant actions to the user. For example, if the system knows the user’s blood glucose level and BMI, it could suggest the number of steps and walking speed required for the user to normalize the blood glucose level based on their historical data. Therefore, such intelligent systems can potentially have a profound impact in transforming the traditional approach of patient monitoring.

The proposed intelligent feedback system can track the user’s gait and PPD in real-time and suggest corrective measures to improve gait and foot health. This system can use a graphical user interface on a smartphone or tactile stimuli such as the vibration of a phone to provide user feedback. The user can then use the feedback to correct themselves or modify behaviors that would otherwise increase their fall risk. Such an intelligent biofeedback system can also decrease an individual’s fear of falling by providing older adults with a sense of ease when engaging in daily activities.

**Figure 13:**
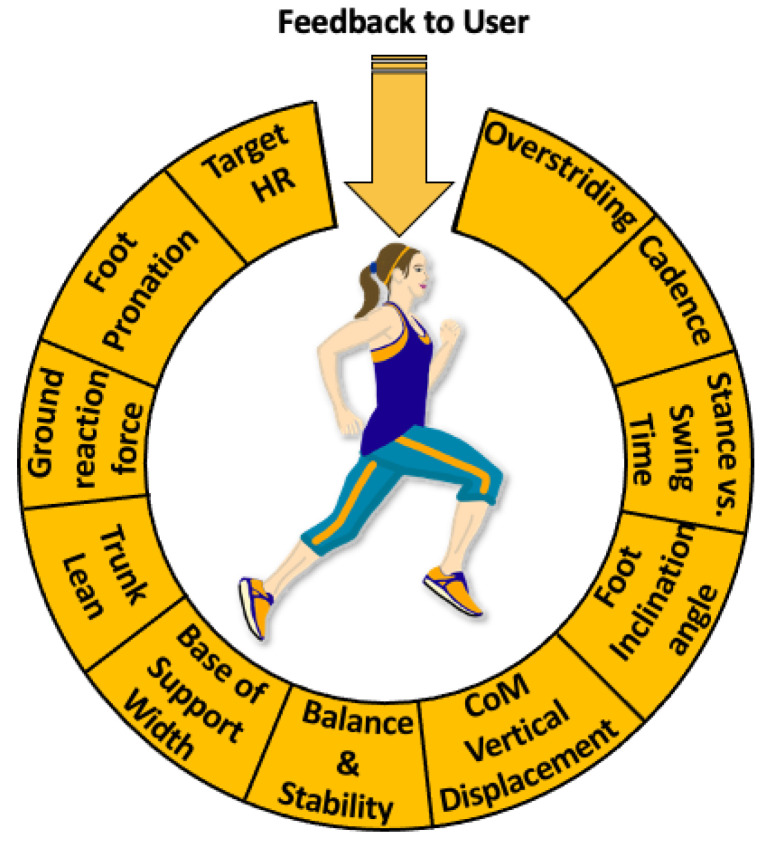
Characteristics which can be addressed when providing biofeedback to a runner in order to reduce the risk of injury when running, in addition to monitoring and improving running performance and health.

The biofeedback system can be very useful for athletes to minimize the risk for injury as well as to improve their athletic performance. Monitoring the gait of a runner can provide insight into healthy running patterns, while also allowing the runner to monitor their physical performance and physiological data, such as step count or heart rate, respectively. Figure 13 outlines some key parameters which may be useful to a runner to reduce the risk of injuries and improve running efficiency. These parameters can be monitored using an insole-based sensor system, and the biofeedback system can be used to allow a runner to monitor and assess their performance and health, while taking corrective actions as appropriate. These various parameters can be measured using a variety of sensors such as pressure sensors, inertial measurement units and heart rate sensors.

Additionally, the data should be easily viewable and understandable to the users of the device and other authorized individuals such as caregivers and physicians. As such, it is important to develop a user-friendly GUI for insole-based systems to facilitate real-time feedback to users. The GUI may be further customized to serve the targeted user. For instance, a simple GUI, with larger fonts and greater contrasts can present a summary report to the users of the older age group. In addition, a detailed report along with measurements can be relayed to and displayed on the interface at the physician’s end.

#### 5.2.3. Use of Smart Textiles

Textile-based sensors or smart textiles have great potential in realizing unobtrusive insole-based monitoring systems. For example, textile-based electrodes and temperature sensors can be used for measuring pulse and insole temperature, respectively. Furthermore, textile-based strain sensors can be exploited for monitoring pulse, foot plantar pressure, gait pattern as well as activities of daily living. Smart textiles can be used to develop smart-socks that would ensure a superior and more stable sensor-skin contact and potentially enable measurement of gait dynamics, plantar pressure, pulse and temperature with higher accuracy. Nonetheless, challenges for a textile-based system include ensuring there is high sensitivity, SNR, signal accuracy and stability. Further research is also needed regarding ideal embedding techniques and the choice of ideal sensing materials to ensure a stable and good sensor performance. In addition, over time and after washing the material, the integrity of the signal and durability of the sensors are also areas in need of further improvement prior to using smart textiles in insole-based systems.

#### 5.2.4. Interdisciplinary Collaboration

A fully functional insole-based monitoring system will ideally be able to analyze, interpret and evaluate the incoming signals and monitor the user’s health in terms of mobility, cardiac activity, and PPD. Therefore, the design of an unobtrusive insole-based monitoring system would understandably require expertise both in software design as well as designing electronic and mechanical hardware. However, expert opinions from health professionals and kinesiologists would be indispensable during the development phase to interpret the sensor signals and validate the effectiveness and accuracy of the system’s assessment. Furthermore, collaborations with hospitals, long-term care centers, and clinics may be useful in receiving feedback about the ease of use and effectiveness of such systems. In addition, collaborations with local shoe manufactures may enable rapid penetration of such systems in the market.

## 6. Conclusions

In this paper, we have presented a state-of-the-art survey on insole-based lower-limb health monitoring systems. The main purpose of such a system is to allow people to remain physically active in their natural day-to-day settings while ensuring continuous and unobtrusive monitoring of lower-limb health. The enormous advancement in sensor technology, coupled with present-day high speed and low-power communication and computing technologies have paved the way to realizing an affordable and non-obtrusive wearable monitoring tool in an insole.

Insole-based systems can facilitate measuring several parameters associated with lower-limb health such as foot plantar pressure, temperature, pulse rate, and gait dynamics. Continuous monitoring of these parameters may potentially have significant implications in enabling remote monitoring of lower-limb health, which is a key health concern particularly for the aging demographic that affects their mobility, activities of daily living, and overall well-being. Such wearable systems would allow monitoring lower-limb health in an individual’s day-to-day activities, thus allowing the user and healthcare providers to obtain and assess information that is reflective of an individual’s health and well-being in their natural settings. Such systems coupled with present-day computing and machine-learning technology can enable early detection of diseases or abnormal gait patterns that can have profound effects on an individual’s health and lifestyle.

By developing a device that is capable of measuring PPD, inertial motion, foot temperature and heart rate, a holistic understanding of an individual’s health and well-being during daily activities can be obtained. Such a multi-sensor monitoring tool can be further coupled with an intelligent system that can exploit machine-learning techniques to perform trend and predictive analysis of health parameters, as well as enable automatic detection of anomalous health patterns. The system can also assess gait patterns for potential health risks such as slips or falls, and make recommendations to the users so as to reduce the fall risk and can automatically raise an alarm in the form of text messages or voice call to alert the user or caregivers in case of an anomaly or medical emergency such as when a fall is detected. However, further research and technology development is required to address the challenges associated with the insole-based health monitoring system that includes determining the optimal number of sensors and their placements, ensuring user comfort and ease-of-use, power efficiency and data privacy and security.

## Figures and Tables

**Table 1:** Key points pertaining to various existing insole-based sensor systems used for plantar pressure measurements and gait analysis.

Author(s) (Year) [Ref]	Sensor Types	Number of Sensors	Sensor Placement	Performance Characteristics	Strengths	Limitations
Guo et al. (2012) [69]	Ceramic piezoelectric pressure sensors	24	8 measuring points: Heel: 3 Forefoot: 5	Wireless data transmission 24 h operation Linear output 100 m maximum transmission distance	3D measurements Small size and mass 24 h data recording	Sample size for validation not large or diverse (3 males)
Hughes et al. (2018) [71]	Soft-strain (conductive silicon) pressure sensor	Variable (application-dependent)	Variable (application-dependent)	Wireless data transmission Range of sensitivity: variable Breathing rate sensor accuracy: 1 bpm (beat per minute) Gait sensor accuracy deemed comparable to existing devices 2.5% error during running and 1% error during walking (750 steps)	Versatile (can measure various parameters) Flexible sensors Low error Fast fabrication (4 h) Customizable	Small sample size Further testing required for sensor under varying circumstances (e.g., with changes in activity or posture)
Lou et al. (2017) [72]	Piezoresistive pressure sensors (Flexible, Graphene-based)	14	Toes: 5 Metatarsals: 5 Lateral midfoot: 1 Heel: 3	Wireless data transmission Measures up to 800 kPa pressure 150 m transmission distance	Flexible Measures up to 800 kPa Linear response Rapid response time	Variability of supporting materials’ shape affects graphene sensor structure
Motha et al. (2015) [73]	Interdigitated capacitive pressure sensors encapsulated in PDMS	3	Forefoot, Midfoot, and Hindfoot	Wired data transmission	Lightweight	Limited regions of foot investigated Wired data transmission Pressure range not suitable (too low and narrow) for plantar pressure applications
Chandel et al. (2019) [74]	Piezoelectric pressure sensors & IMU	5 piezoelectric + 1 IMU	Interphalangeal joint, Metatarso-phalangeal joint, Heel, Lateral calcaneus, Distal metatarso-phalangeal joint; IMU at midfoot	Wireless data transmission 94.5% accuracy for calculating duration of swing/stance phases 2.8 cm error in stride length calculation	Includes IMU 3D stride trajectory tracking	Limited number of foot regions investigated
Martini et al. (2020) [75]	Optoelectronic pressure sensors	16	Forefoot: 8 Midfoot: 3 Hindfoot: 5	Wireless data transmission Accuracy comparable to other existing devices Insole’s overall mean absolute error of heelstrike = 6.47%; toe-off = 4.32%.; stance duration = 2.02%	Clustering of sensors: redundancy	Limited areas of foot examined
Saito et al. (2011) [76]	Pressure-Sensitive Conductive Rubber (PSCR) sensors	7	Hallux, Heel, Central midfoot, Lateral midfoot, Central forefoot, Lateral forefoot, First metatarsal	Wireless data transmission 25 to 250 kPa range 20 h battery life Maximum error across measurement range: 15%	Smaller data transmission unit than is typical 20 h battery life	Pressure range may not be suitable for high-impact activities User-specific calibration required Small sample size for validation High cost 10 m maximum transmission distance
Sorrentino et al. (2020) [77]	Capacitive pressure sensors (grouped into modular units)	336 Pressure:280 Temp:56 Grouped into 28 modules	Nearly entire foot (arranged in triangular modular units)	Wired data transmission Similar accuracy as commercial force-torque sensors	Large number of sensors High spatial resolution	Wired data transmission

**Table 2:** Key points pertaining to various insole-based and related sensor systems which have incorporated triboelectric nanogenerators.

Author(s) (Year) [Ref]	Application	Sensor Types	Number of Sensors	Sensor Placement	Performance Characteristics and Other Information
Lin et al. (2019) [83]	Gait monitoring (distinguish activities such as jump, walk, run)	Triboelectric sensor [Two parts: convex TENG portion + elastic air chamber (EAC)]	Two	At forefoot (beneath medial metatarsal heads) and hindfoot	Real-time gait monitoring Fast response time (less than 56 ms) Durable Mechanically robust Negligible decrease in electrical output over 1000 cycles Layers: - Convex rubber film (20 mm diameter) at top - Integrated copper layer (shields against environmental interference) - Additional copper film (bottom layer of TENG) - Supporting acrylic layer - Stretchable latex film for Elastic air chamber (to temporarily retain air from TENG)
Huang et al. (2021) [84]	Plantar Pressure Monitoring	Piezoresistive and Triboelectric nanogenerator	31 piezoresistive (each sensor’s size: 7.5mm × 7.5 mm) TENG included in shoe-pad	Across entire foot	Fast acquisition of data from pressure matrix High-speed communication Pressure sensing range: 20 pa to 1.2 MPa 100 ms response time 0.5 Hz to 2.0 Hz frequency range 500 s for TENG to charge 4.4 uF capacitance Wireless data transmission Continuous operation Self-powered TENG provides continuous power supply Lightweight Low power data acquisition Frequency range encompasses normal walking frequency Real-time data Low cost Limited testing on human subjects reported
Deng et al. (2018) [85]	Plantar Pressure Mapping	Piezoelectric nanogenerators Triboelectric-electromagnetic nanogenerator	32 (PVDF-based PENG pressure sensors) Hybridized triboelectric electromagnetics nanogenerator included	Sensors placed at forefoot, lateral midfoot and hindfoot	Wireless data transmission Real-time pressure mapping Self-powered Low cost Pressure range up to 200 kPa Pressure sensitivity reflected by free charges: 23.4 pC N^−1^ Low current required for data acquisition system
Tao et al. (2021) [86]	Plantar pressure mapping and Small aerial vehicle flap motion energy harvesting	Honeycomb-inspired triboelectric nanogenerator (h-TENG) Arch-shaped capacitors in parallel-connected array	Ten h-TENG devices (bound to flexible printed circuit board) Each h-TENG unit’s volume = 20mm × 15mm × 10 mm (for insole application)	Across sole	h-TENG instantaneously produces: - Open-circuit voltage: 1207 V - Short-circuit current: 68.5 μA - Output power: 12.4 mW Peak power density: 2.48 mW g^−1^ Flexible Lightweight Porous honeycomb structure allows for several energy generation units Elastic and self-rebounding properties of honeycomb structure
Somkuwar et al. (2020) [87]	Textile-based applications	Woven TENG	Varying woven TENG unit structures	Part of fabric (e.g., sock material at plantar aspect of foot)	Greatest average power density = 12.84 μW/cm^2^ (using 5/1 twill) Higher electrical output using 2/2 matt and 3/1 twill weave (compared to 1/1 plain weave) Foldable, twistable, stretchable Flexible Fabricated from basic weaving (no treatments) Increasing contact area led to greater charge induced on triboelectric
Zhu et al. (2019) [88]	Gait sensing, Motion tracking, Walking pattern recognition, Sweat sensing	TENG textile integrated with lead zirconate titanate (PZT) piezoelectric sensor coated with PEDOT:PSS (poly(3,4-ethylenedioxythiophene) polystyrene- sulfonate)	Five coated sensing areas	Coated sensing areas: hallux, forefoot, heel, left instep, right instep	Self-powered Wireless Cotton sock PZT chips used (20 μm PZT chip laser cut to 5mmx5mm) Sensitivity: 0.06 V/N Triboelectric output power: 1.71 mW (power density = 11 μW/cm^2^) PZT piezoelectric power density is 128 μW/cm^2^ As 0.9g sweat absorbed, 80% decrement of output voltage obtained
Zhang et al. (2020) [89]	Gait analysis and Virtual reality applications	T-TENG (textile-based TENGs)	1 (similar size of user’s foot)	Beneath entire plantar aspect of foot	Self-powered from 1 Hz walking on load of 44.4 MΩ, 0.32 mW output power generated from running (2Hz) on 21.3 MΩ load, maximum output power = 3.18 mW Pressure sensing range beyond 200 kPa (acceptable for plantar pressure detection) Sock can charge up to 27 μF in 3–4 min Deep learning model optimized for gait analysis 96.67% accuracy of human activity detection (among 5 activities and 13 participants) Low cost Wireless

**Table 3:** Key points pertaining to various existing insole-based sensor systems used for gait and activity monitoring.

Author(s) (Year) [Ref]	Sensor Types	Number of Sensors	Sensor Placement	Performance Characteristics	Strengths	Limitations
Jung et al. (2013) [98]	Pneumatic pressure sensors and IMU	5 (Pressure: 4, IMU: 1)	Pressure sensors: Toe: 1; Metatarsals: 2; Heel: 1 IMU: behind the foot	Wireless data transmission Data sample rate: 100 Hz	Ground reaction force (GRF) and foot orientation measurement Wireless data transmission	Limited regions of foot investigated Sufficient experimental data are not reported
Wang et al. (2015) [90]	Resistive pressure sensors and IMU	10 (Pressure: 8, IMU: 1)	Pressure sensors: Toe: 1; Metatarsals: 3; Lateral midfoot: 1; Heel: 3 IMU: on top	Wireless data transmission Over 24 h operation Force range: 0–100 lbs.	3D measurements Long data transmission range (>20 m) High data sampling rate (>1 KHz for pressure sensors and ~100 Hz for accelerometer and gyroscope) Long recording time (24 h) with on-board SD card	Limited regions of foot investigated Sampling frequency of the IMU is limited to 100 Hz
Mustufa et al. (2015) [91]	Piezoelectric sensors, IMU, temperature sensor and force sensor	35 (Pressure: 32, IMU: 1, Temp: 1, Force: 1)	Pressure sensors: uniformly distributed at 32 points across the insole area IMU at Heel	Wireless (Bluetooth) data transmission 120 min of continuous operation (280 mAh Li-ion battery) Pressure range of 15 kPa–1000 kPa Temperature sensor records ambient temperature of smart insole Force sensor is used for automatic activation of the insole	3D motion capture (can support up to ±6 g) High sensitivity of the pressure sensors (0.5% of the full-scale range) low cost and scalable (200 nodes can be accommodated in pressure sensor array) Can measure ambient temperature	Limited operation time (continuously operate for only 120 min)
Lin et al. (2016) [92]	Piezoelectric sensors, IMU	49 (Pressure: 48, IMU: 1)	Pressure sensors uniformly distributed 48 sensors array across the insole area IMU at Heel	Wireless (BLE) data transmission 24 h operation Range of pressure: 30–1200 kPa Range: Accelerometer: ±16 g, Gyroscope: ±2000 °/s 256-kB in-system programmable flash memory	Low-cost, lightweight, thin, and comfortable to wear 3D measurements Wireless data transmission 24 h working duration High pressure range (30–1200 kPa) with low response lag (<5%)	A robust computational model is required for energy expenditure calculation A larger cohort study is needed to prove wearability and usability of the system
Jagos et al. (2017) [93]	Resistive pressure sensors and IMU	5 (Pressure: 4, IMU: 1)	Pressure sensors: Toe: 1; Metatarsals: 2; Heel: 1 IMU at midfoot	Wireless (Bluetooth) data transmission Pressure sensor range up to 31,138 N (7000 lb) Li-Po battery (150 mAh)	3D motion capture Wireless data transmission On-board microSD memory card	Limited regions of foot investigated Small battery life (150 mAh)
Roth et al. (2018) [94]	Resistive pressure sensors and IMU	4 (Pressure: 3, IMU: 1)	Pressure sensors: Metatarsals: 2; Heel: 1 IMU at midfoot	Wireless (BLE) transmission Pressure range: 0.1–100 N Li-po battery (120 mAh) with 40h run-time Sample logging frequency: 200 Hz	3D motion capture Wireless data transmission On-board flash memory (4 Gbit)	Limited regions of foot investigated Small battery life (40 h) Mean error: 0.064 ± 0.06 (Double support time), 3.89 ± 2.61 (% of Double support)
Refai et al. (2018) [96]	Resistive pressure sensors, 3-D forces and moments (F&M) sensors, IMU and ultrasound sensors	156 (Pressure: 151, F&M: 2, IMU: 2, Ultrasound: 1)	Pressure sensors: uniformly distributed across the insole area F&M sensors: Forefoot: 1; Hindfoot: 1 IMU: Forefoot: 1; Hindfoot: 1 Ultrasound at toe	Wireless (Bluetooth) transmission Sensors were synchronized Data sample rate: 50 Hz	3D motion capture Wireless data transmission Synchronized data collection	External wireless transmitters need to be worn as a belt around the waist. Heavy and Bulky. The shoe weight was almost 1kg with increased sole height by 2.5 cm Mean abs. rms error of extrapolated CoM (XCoM): 2.2 ± 0.3 cm
Choi et al. (2018) [102]	Pressure sensors, accelerometer and Gyroscope	10 (Pressure: 8, Accelerometer: 1, Gyroscope: 1)	Pressure sensors: Toe: 1; Metatarsals: 5; Heel: 2 IMU at midfoot	Wireless (BLE) transmission Data sample rate: 100 Hz Flash memory Wireless charging	3D motion capture Wireless data transmission On-board flash memory Wireless charging Personal mobile gait analysis system named “fGait”	Limited regions of foot investigated For stair ascending (SA) and stair descending (SD) only 1-min data was collected
Djamaa et al. (2020) [105]	Resistive pressure sensors, Resistive bend (flex) sensors, and IMU	5 (Pressure: 3, Flex: 1, IMU: 1)	Pressure sensors: Toe: 1; Metatarsals: 1; Heel: 1 Flex sensor at midfoot IMU: behind the foot	Wireless (BLE) transmission The prototype was made using Arduino UNO	Low-cost and lightweight 3D measurements Wireless data transmission	Limited regions of foot investigated
Farid et al. (2021) [106]	Pressure sensors and IMU	5 (Pressure: 18, IMU: 1)	Pressure sensors: Forefoot: 14; Heel: 4 IMU at midfoot	Wireless (Bluetooth) transmission Wireless (inductive) charging Internal data storage (up to 26 days movement data) 12 sizes	Thin, lightweight and comfortable 3D motion capture Store up to 26 days movement data Wireless data transmission Wireless charging Medical CE/FDA approved Various sizes are available	Plantar pressure of midfoot region cannot be investigated Large charging time (180 min for full charging) Small battery life (110 mAh) Home use was not evaluated Error: 3.1–4.7% in comparison to an odometer
Duong et al. (2020) [107]	Resistive pressure sensors and IMU	9 (Pressure: 8, IMU: 1)	Pressure sensors: hallux, toe, first/third/fifth metatarsals, lateral arch, and medial/lateral calcaneous IMU at midfoot	Wireless (Wi-Fi) transmission Data transmission rate: 500 Hz Insole thickness: ~5.5 mm Total weight: ~50g	Thin, lightweight, and comfortable 3D motion capture Wireless data transmission Various sizes (4) are available	Designed for only toddlers’ and children’s shoes (3 to 12 years)
Chen et al. (2020) [109]	Piezoresistive pressure sensors and IMU	97 (Pressure: 96, IMU: 1)	Pressure sensors: uniformly distributed across insole IMU at midfoot	Wireless (Bluetooth) transmission Data sampling rate: 30 Hz Built-in Li-ion battery (3.7 V, 1000 mAh)	3D motion capture Wireless data transmission Large rechargeable battery shows the visualization of the plantar pressure on the smartphone application	Low sampling frequency (30 Hz) Complex real-world conditions are not analyzed.

**Table 4:** Key points pertaining to various existing sensor systems measuring physiological parameters at or near the foot.

Author(s) (Year) [Ref]	Focus of Work	Sensor Type/System	Number of Sensors Per Foot/Leg	Sensor Placement	Other Useful Information
Jarchi and Casson (2016) [111]	Heart Rate	Photoplethysmography	1 PPG sensor	At ankle	Energy harvesting capabilities 9 bpm error
Diaz et al. (2010) [112]	Heart Rate	Impedance Plethysmography—IPG	7 (injecting and detecting) electrodes	Beneath plantar aspect of foot (in weighing scale)	Impedance suitable for leg amputees, pregnant women, electronic implant users and pacemaker users
Liu et al. (2017) [16]	Heart Rate	Ballistocardiography—BCG (polyvinylidene fluoride film sensor)	1	Beneath plantar aspect of foot (in insole-based system) and in seat cushion (at thighs)	Weak signals obtained unless cardiac output enhanced following exercise
Zhang et al. (2019) [113]	Heart Rate and Muscle Activity	Iontronic capacitive pressure	5 sensing channels	Dorsal aspect of foot (dorsalis pedis arterial pulse)	Device capable of detecting both heart rate and muscle activity from dorsal aspect of foot
Hong and Park (2018) [17]	Heart Rate	Photoplethysmography—PPG	4 LEDs and 30 photodetectors	Beneath plantar aspect of foot (in insole-based system) Targeting lateral plantar artery	Stable signals obtained only when standing still
Isezaki et al. (2019) [19]	Muscle Activity	EMG electrodes	10 conductive-fabric electrodes	At calf (in sock system)	Sock-based system allows for electrodes to avoid slipping from appropriate position
Wu et al. (2020) [114]	Temperature and Pressure	Temperature: Thermistors Pressure: printed side-by-side electrodes	4 thermistors 12 pressure sensors	Beneath plantar aspect of foot (in insole-based system)	Pressure range: 7.4 Pa to 1 million Pa

**Table 5:** Key points pertaining to various activity and gait analysis systems using advanced machine learning techniques.

Author(s) (Year) [Ref]	Objectives of the Study	Sensor Types	Methods of Analysis	Measured Parameters and Outcomes
Muniz et al. (2006) [140]	Determining the feasibility of PCA in the ground reaction force (GRF) data to distinguish the normal and abnormal gait patterns for the rehabilitation treatment	Instrumented treadmill Gaitway^®^ model 9819S1 with force platform	PCA, LR	PCA is useful to categorise (scatter diagram of first two coefficients) between normal and abnormal gait. However, only 48.9% data variance was reported in this study between controlled and patient groups
Lai et al. (2007) [141]	Classification of gait patterns between patients with patellofemoral pain syndrome (PFPS) and healthy group using SVM	Four video cameras (Panasonic WV-CL830/G colour CCTV) 10 m walkway with an embedded force platform	SVM	Classification accuracy (using polynomial kernel) was 85.19% for 14 GRF feature set while for 16 kinematics features the accuracy was 74.07% Using optimal feature set (six) from both GRF and kinematic data, SVM achieved the highest accuracy of 88.89%
Lau and Tong (2008) [142]	Evaluation of accelerometer and gyroscope-based system for gait event identification	3 Sensor units (accelerometer and gyroscope) at thigh, shank, and foot 4 FSRs at 2nd middle phalanx, 1st and 5th metatarsal head, and heel	Threshold detection method	Turning points (maximum/minimum) Classification of the detected turning points Identification of gait events
Alaqtash et al. (2011) [143]	Classification of gait patterns of healthy, cerebral palsy (CP) and multiple sclerosis participants using 3D GRFs data	Instrumented treadmill with two independent force plates mounted underneath the belts	DWT, ANOVA, NNC, and ANN	GRF parameters using NNC achieved higher accuracy rate than DWT Both NNC and ANN achieved the same range of classification accuracy: 85% without feature selection and 95% with optimal feature set (six features)
Jung et al. (2012) [144]	Detection of stroke patients’ intention (desire to move and time) for exoskeleton robots during rehabilitation	Ground reaction force sensor Joint angles measurement unit (not specified)	NN	Classification of one stride into seven sub-phases using binary representation. Gait pattern generation for the affected leg.
Guo et al. (2017) [145]	Classification of normal and pathology-related changes in gait using foot pressure data in young children	GAITRite Electronic Walkway	PCA, Discriminative mapping, SVM, and RF	SVM achieved 94.36% and and RF achieved 97.50% classification accuracy based on age information and other spatiotemporal features. Demonstrated the possibility to distinguish minimal variations during early stages of gait development such as changes in foot shape in young children
Jiang et al. (2018) [146]	Gait patterns recognition for rehabilitation therapy	Insole-based graphene porous network structure pressure sensors (GPNSPS)	Ensemble learning framework - SVM + RF + LR	Detection of different gait patterns (accuracy) - Normal gait (94.3%) - Toe in (83.1%) - Toe out (94.2%) - Lame (93.6%) - Heel (96.8%)
Nazmi et al. (2018) [147]	Classification of gait phases such as stance and swing using surface EMG (sEMG)	sEmg attached on tibialis anterior (TA) and medial gastrocnemius (mGAS) muscles Two FSRs placed at the hallux and heel under the sole of foot	ANN with LM	Classification of stance and swing phase with accuracy: - 87.5% (learned data) - 77% (unlearned data)
Souza and Stemmer (2018) [148]	Different movement patterns extraction for gait analysis and a comparative study of different pattern recognition techniques for human identification	Microsoft Kinect sensor	SVM, MLP, RF, PNN, Naive Bayes, LVQ, kNN, and DNN	PNN, DNN, and kNN presented the highest classification accuracy in the 99% range - DNN had the highest training time of 99.51 s while kNN had the lowest of 0.63 s SVM, MLP, NB, and RF had the accuracy in the 97% range LVQ showed the lowest accuracy of 36%
Huang et al. (2018) [149]	Modeling the control system of lower exoskeleton for hemiplegia patient with a Leader-Follower Multi-Agent System (LF-MAS) applying reinforcement learning framework	Joint actuators IMU sensors Plantar sensors placed in soles	PI-ADP algorithm	LF-MAS based on a lower exoskeleton system (AIDER) presented good performance with healthy subjects
Zhang and Ma (2018) [150]	Evaluation of supervised machine learning algorithms to classify sagittal gait patterns of cerebral palsy (CP) children with spastic diplegia	Eight camera-based motion analysis system (Vicon MX, Oxford Metrics, UK) Three force platforms	ANN, discriminant analysis, Naive Bayes, decision tree, kNN, SVM, and RF	Predictive accuracy and resubstitution error: - ANN: 93.5%, 5.8% - SVM: 85%, 5.7% - Decision tree: 84.3%, 5.7% - Discriminant analysis: 84.3%, 14.3% - RF: 83.6%, 6.4% - Naive Bayes: 72.1%, 13.6% - kNN: 77.9%, 0%
Cui et al. (2018) [151]	Development of an automatic gait analysis system to recognize and evaluate abnormal gait among the post-stroke hemiparetic patients	A six camera-based Qualisys motion capture system Two Bertec force plates Eight channels of surface EMG	SVM, NN, RF, Naive Bayes, and KNN Fusion algorithms: AR and MR	Classification performance: - Single modal—RF: 92.26% (based on GRF data) - Two modal—SVM: 95.83% (based on marker trajectory and GRF data) - Three modal—SVM: 98.21% (based on marker trajectory, GRF, and EMG data) Proposed walking ability mean score (WAMS) has the potential in clinical application to quantify the differences between pathological and normal gait
Sobral et al. (2018) [152]	Development of gait indices—NGI and AGI (normal and abnormal gait index) to evaluate the recovery status after anterior cruciate ligament (ACL) Reconstruction	Eight insole-based force sensors	ELM	Developed gait indices discriminated healthy and impaired gait patterns based on symmetry and measured Gait error (calculated from deviations between 16 parameters from Vertical GRF) Ideal gait is supposed to have Gait error and symmetry index equal to zero
Thongsook et al. (2019) [153]	Gait phases (Stance, swing, and push) recognition for lower limb exoskeleton	Two IMU sensors at hip and knee joints Two FSRs placed at toe and heel	C4.5 decision tree, MLP, and NARX	In gait phase recognition, C4.5 decision tree performs better (100% accuracy) than MLP (94.79%) and NARX (98.76%) with larger training dataset (≤10,000)
Zhang et al. (2020) [154]	Extraction of fundamental gait parameters such as stride length, velocity, and foot clearance accurately during walking and running tasks	A multi-cell piezo-resistive sensor IMU	SVR	2-step calibration method - zero velocity update and velocity drift compensation - SVR-based gait parameter estimation based on intraclass correlation coefficients (ICC)
Tong et al. (2021) [155]	Development of a method based on permutation-variable importance (PVI) and persistent entropy to classify the severity classification of Parkinson’s disease (PD) patients	16 Insole-based force sensors (eight sensors under each foot)	RF, SVM	RF was used to separate the leading factors distinguishing the gait of patients at different severity levels SVM classification achieved an accuracy of 98.08% by 10-fold cross-validation

NN: neural network; SVM: support vector machine; RF: random forest; LR: logistic regression; ANN: artificial neural network; LM: Levenberg–Marquardt; MLP: multilayer perceptron neural network; PNN: probabilistic neural networks; LVQ: learning vector quantization; kNN: k-nearest neighbors; DNN: deep neural networks; PI-ADP: policy iteration adaptive dynamic programming; NARX: nonlinear autoregressive with exogenous variable; PCA: principal component analysis; DWT: discrete wavelet transform; ANOVA: analysis of variance; NNC: nearest neighbor classifier; AR: average rule; MR: max rule; ELM: extreme learning machine; SVR: support vector regression.

## Data Availability

Not applicable.
